# The cumulative incidence and trends of rare diseases in South Korea: a nationwide study of the administrative data from the National Health Insurance Service database from 2011–2015

**DOI:** 10.1186/s13023-019-1032-6

**Published:** 2019-02-18

**Authors:** Sung-Shil Lim, Wanhyung Lee, Yeong-Kwang Kim, Jihyun Kim, Jong Heon Park, Bo Ram Park, Jin-Ha Yoon

**Affiliations:** 1 Graduate School of Public Health, Yonsei University, Seoul, Republic of Korea; 2 The Institute for Occupational Health, Yonsei University College of Medicine, Seoul, Republic of Korea; 3 Department of Occupational and Environmental Medicine, Seoul St. Mary’s Hospital, College of Medicine, The Catholic University of Korea, Seoul, South Korea; 4 Department of Big Data Steering, National Health Insurance Service, Seoul, South Korea; 5 Department of Preventive Medicine and Public Health, Yonsei University College of Medicine, Seoul, Republic of Korea

**Keywords:** Rare disease, Incidence, Administrative data, Population-based

## Abstract

**Background:**

The burden of rare diseases on society and patients’ families has increased in Korea. However, because of the infrequency of rare diseases, there is a lack of resources and information to address these cases and inadequate funding for the management of these patients. We investigated the average annual cumulative incidence of rare diseases and the trends in annual cumulative incidence from 2011 to 2015 in Korea by using nationwide administrative data from the Korean National Health Insurance Service (NHIS) database for patients registered with the co-payment assistance policy for rare and incurable diseases. Annual cumulative incidence per 10,000,000 was calculated as the total number of newly enrolled patients with the Korean Standard Classification of Diseases (KCD)-7 code in the register, divided by the number of residents with health insurance coverage during each year. We employed simple linear regression analysis to evaluate the trends in annual cumulative incidence/10,000,000 population per year for each rare disease.

**Results:**

Overall, national support was provided for patients with 415 KCD codes listed among the targeted rare diseases. The total number of newly enrolled patients with rare diseases was 53,831 in 2011, 52,658 in 2012, 52,955 in 2013, 71,530 in 2014, and 70,559 in 2015. The number of rare diseases with an average annual cumulative incidence of 100/10,000,000 and above was 22 (5.30%), while there were 227 (54.70%) and 148 (35.66%) with an average cumulative incidence between 1/10,000,000 and 100/10,000,000 and less than 1/10,000,000, respectively. The trends in the annual cumulative incidence for 43 rare diseases were statistically significant (*p*-value < 0.05). The rare diseases for which the incremental trend per year was statistically significant were sarcoidosis (D86, D86.0, D86.1, D86.2, D86.3, D86.8, D86.9), Parkinson’s disease (G20), Guillain-Barré syndrome (G61.0), primary biliary cirrhosis (K74.3) and Sjogren’s syndrome (M35.0).

**Conclusions:**

The number of rare diseases showing an increasing trend in annual cumulative incidence was higher than the number of diseases showing a decreasing trend in annual cumulative incidence. Given that the definition and diagnosis vary based on country and that there is difficulty in identifying valid cases, further detection strategies are needed to establish the incidence of each rare disease considering the importance of establishing a health policy based on the actual incidence of the targeted diseases.

**Electronic supplementary material:**

The online version of this article (10.1186/s13023-019-1032-6) contains supplementary material, which is available to authorized users.

## Background

A rare disease is defined as any disease with a low prevalence. However, the exact definitions of rare diseases vary from country to country. In the European Union, a rare disease is defined as one that affects less than 1 in 2000 [1]. A disease is defined as rare in the United States of America when it affects fewer than 200,000 people at any given time [2]. In South Korea, the term rare disease applies to diseases for which there are fewer than 20,000 patients, or for which the prevalence is unknown owing to difficulties in diagnosing the disease or that are designated by the procedures and standards set by the Ministry of Health and Welfare [3]. Despite the low prevalence of each rare disease, the burden of rare diseases on patients’ families and society is considerable owing to the chronicity and relatively incurable pattern of rare diseases [4]. According to the report from the Commission of the European Communities in 2008, it is estimated that 30 million European Union citizens have rare diseases [5]. In South Korea, the patient cost of care for rare diseases has increased. The total cost of medical care benefits for rare and intractable diseases increased from 2.6 billion US dollars in 2010 to 4.1 billion US dollars in 2016 [6]. Due to the chronicity of several rare diseases and the high economic cost of treatment, patients with rare diseases need social support. However, considering the infrequency of rare diseases, affected patients are likely to be neglected, resulting in a lack of resources and information to address these cases and inadequate funding for the proper care of these patients [7]. Furthermore, many patients with rare diseases would have visited several clinics or hospitals, but would only receive symptomatic treatment, owing to the lack of knowledge about the pathophysiology of rare diseases and the difficulties in their proper diagnoses [8–11]. After being correctly diagnosed with a rare disease, affected patients may not receive proper care because of the lack of adequate treatment or high price of medication (“orphan drugs”) [4, 12, 13].

South Korea introduced a legislation regarding orphan drugs in 2003 and regarding the management of rare diseases in 2016 [3, 14]. Even before the introduction of the rare disease management act, the government had subsidized medical expenses for patients with rare and intractable diseases through a co-payment assistance policy that was in place since 2001. The targeted rare diseases covered by the policy (see Additional file 1) are determined via expert consultation based on their rarity and severity. In order to benefit from national support, the disease must be registered in the register of the co-payment assistance policy, which is supported by the National Health Insurance Service (NHIS). The entire population residing within the territory of Korea is covered by the National Health Insurance (NHI) of Korea [15]. Registered patients with rare diseases make out-of-pocket payments that comprise about 10% of the total cost of medical treatment, which is normally 20–60% of the total cost of treatment. Furthermore, patients with some rare diseases receive support for the purchase of assistive devices such as those for the disabled, respiratory aids, and cough inducers, as well as support towards the cost of caregiving [3].

The investigation of the incidence and prevalence of diseases should be preceded by establishing the public health policy for the specific disease. However, investigators have faced difficulties in studying the epidemiology of rare diseases because of difficulties in detecting the few existing cases [16]. Several studies have attempted to understand the epidemiology of rare diseases. For example, Seo et al., using NHIS data, reported that the prevalence of amyloidosis was 0.93 (95% confidence interval [CI]: 0.81–1.04) in Korea [17]. Ryder et al. reported that the point prevalence of Duchenne muscular dystrophy per 100,000 men in France, the USA, UK, and Canada was 10.9, 1.9, 2.2, and 6.1, respectively [18]. Groth et al. showed that the annual median incidence of Marfan syndrome was 0.19/100,000 population (range: 0.0–0.7) [19]. Furthermore, Orphanet conducted a systemic review of literature to estimate the incidence and prevalence of rare diseases [20]. However, most of the studies did not use the rare disease register data, but rather the medical claim data [17, 18]; thus, it is not easy to obtain the real incidence of each rare disease [21]. Therefore, population-based administrative records of rare disease registries are appropriate sources of data to conduct a passive surveillance of rare diseases to maximize valid case detection [21].

To investigate the population-based annual cumulative incidence (2011–2015) of rare diseases in Korea according to the Korean Standard Classification of Diseases (KCD) [22], we used the administrative data from the register of the co-payment assistance policy maintained by the NHIS for rare and incurable diseases in South Korea. We also examined the trends in the annual cumulative incidence of each rare disease from 2011 to 2015.

## Methods

### Study population

We used the administrative data regarding application for registration in the co-payment assistance policy with the NHIS for the period of 2011–2015 in order to determine the population-based incidence of each rare disease in Korea. These data included the information of the applicants, code of the targeted disease classified per the KCD-7 based on the International Classification of Diseases (ICD)-10 [23], date of definite diagnosis and start date of the application for co-payment assistance, and tests performed for the diagnosis of the rare disease. We included all patients who registered for the co-payment assistance policy and actually received national support. Only patients who met the diagnostic criteria which NHIS had defined for each rare disease on the basis of the results of comprehensive medical tests including imaging studies, biochemistry, immunology, smear, culture test, histological examination and clinical diagnosis by the physician could be registered in the system. For example, only patients with Crohn’s disease confirmed by the combined results of imaging studies, clinical evaluation, endoscopy and biopsy are eligible for national support by NHIS. The annual cumulative incidence of rare diseases was calculated using the KCD code in the application. Overall, 415 KCD codes were used to calculate the annual cumulative incidence of each rare disease. We used the year of definite diagnosis by the physician in the application form as the year of disease occurrence. We excluded the data regarding reapplications to extend national support, which occurs every 5 years, thus including data for only the first enrolment for each disease. Finally, we excluded patients with missing values for sex and age.

### Statistical analysis

All residents within the territory of Korea are covered by the NHI of Korea [15]. It is mandatory for all citizens of Korea to be associated with the NHI by law. The number of residents with health insurance coverage was 50,908,646 in 2011, 51,169,141 in 2012, 51,448,491 in 2013, 51,757,146 in 2014, and 52,034,424 in 2015, which represent approximately 98% of the people living in the territory of Korea [24].

Annual cumulative incidence per 10,000,000 was calculated as the total number of newly enrolled patients with the KCD-7 code in the register of the co-payment assistance policy for rare and incurable diseases during a calendar year, divided by the number of residents with health insurance coverage in each year (see Additional file 2). The average annual cumulative incidence was calculated as the mean annual cumulative incidence from 2011 to 2015. However, we calculated the average annual cumulative incidence during the 2 years (2014–2015) of newly enrolled targeted diseases (KCD codes: G40.4, G40.40, G40.41) owing to the expansion of coverage for these diseases in 2014. We employed a simple linear regression analysis to evaluate the trends in annual cumulative incidence/10,000,000 population per year for each rare disease. *P*-values < 0.05 were considered statistically significant. All analyses were conducted using SAS version 9.4 (SAS Institute, Cary, NC, USA) and R (R Development Core Team, Vienna, Austria).

## Results

The total number of newly enrolled patients with rare diseases benefiting from national support was 53,831 in 2011, 52,658 in 2012, 52,955 in 2013, 71,530 in 2014, and 70,559 in 2015. Table 1 shows the average annual cumulative incidence per 10,000,000 of the insured population (2011–2015) with rare diseases and the trends in annual cumulative incidence per year for each rare disease in the register of the co-payment assistance according to the KCD code. The number of rare diseases with an average annual cumulative incidence of 100/10,000,000 and above was 22 (5.30%); the number of rare diseases with an average annual cumulative incidence between 1/10,000,000 and 100/10,000,000 was 227 (54.70%) and that for rare diseases with an average annual cumulative incidence less than 1/10,000,000 was 148 (35.66%) (data not shown in table). There were no reported cases of 18 (4.34%) KCD code diseases during the study period.

**Table 1:** Average annual cumulative incidence/10,000,000 and trend of annual cumulative incidence of rare diseases in the register of the co-payment assistance policy according to KCD codes from 2011 to 2015

KCD	Average annual cumulative incidence^a^	Annual trend (β)^b^	*p*-value
A81	0.31	−0.02	0.898
A81.0	6.48	0.71	0.203
A81.1	0.04	0.04	0.182
A81.2	0.74	−0.16	0.063
A81.8	0.08	0.02	0.646
A81.9	0.12	0.02	0.654
B45	0.35	0.08	0.322
B45.0	7.46	0.37	0.353
B45.1	3.69	−0.10	0.736
B45.2	0.16	−0.08	0.138
B45.3	0.08	−0.02	0.629
B45.7	0.15	0.08	0.141
B45.8	0.15	0.08	0.142
B45.9	1.16	0.23	0.102
D35.2	562.81	12.94	0.366
D55.0	0.86	− 0.24	0.268
D55.2	0.19	0.02	0.818
D56	0.00	.	.
D56.0	0.08	0.02	0.639
D56.1	1.86	0.16	0.491
D56.3	0.19	−0.02	0.704
D56.4	0.08	0.02	0.639
D56.8	0.15	0.08	0.142
D56.9	1.24	0.19	0.430
D59.5	5.82	0.90	0.107
D60	0.35	0.09	0.366
D60.0	0.19	−0.06	0.211
D60.1	0.12	0.02	0.647
D60.8	0.62	0.09	0.701
D60.9	5.86	0.54	0.403
D61.0	3.74	−0.43	0.133
D61.2	0.58	0.10	0.487
D61.3	18.52	1.48	0.090
D61.8	2.53	−0.05	0.803
D61.9	123.60	1.69	0.612
D64.4	0.08	−0.02	0.629
D69.1	3.32	−1.17	0.143
D69.30	5.60	−0.21	0.777
D69.6	99.82	−8.77*	0.046
D70	41.74	−3.29*	0.044
D71	1.72	−0.48	0.236
D76.1	21.40	1.36*	0.012
D76.3	9.39	1.03	0.374
D80	0.16	−0.08	0.305
D80.0	1.01	0.33	0.465
D80.1	1.40	0.17	0.740
D80.2	1.64	−0.34	0.304
D80.3	5.11	1.42*	0.033
D80.4	0.04	0.04	0.182
D80.5	0.12	0.06	0.559
D80.6	0.04	−0.02	0.559
D80.7	0.08	0.08	0.182
D80.8	0.08	0.00	0.984
D80.9	0.23	0.04	0.647
D81	0.00	.	.
D81.1	0.08	0.04	0.311
D81.2	0.04	0.02	0.559
D81.8	0.04	−0.04	0.182
D81.9	0.23	0.00	0.984
D82	0.00	.	.
D82.0	0.31	0.04	0.660
D82.1	2.13	0.65	0.140
D82.3	0.08	0.00	0.984
D82.4	0.31	−0.08	0.322
D82.8	0.04	0.04	0.182
D83	0.00	.	.
D83.0	0.12	0.00	0.996
D83.9	0.31	0.06	0.499
D84	0.04	0.02	0.559
D84.0	0.00	.	.
D84.1	1.32	−0.07	0.752
D84.8	0.08	0.00	0.984
D84.9	0.78	−0.04	0.568
D86	3.81	−0.35	0.282
D86.0	22.89	3.72*	0.003
D86.1	12.46	1.64*	0.027
D86.2	8.96	1.35*	0.017
D86.3	4.61	0.87	0.219
D86.8	4.04	−0.06	0.885
D86.9	26.68	1.56	0.133
E22.0	28.45	3.00	0.318
E23.0	35.21	−0.14	0.958
E24.0	3.84	0.48	0.508
E24.1	0.08	0.00	0.984
E24.3	0.16	−0.04	0.692
E25	0.82	0.07	0.575
E25.0	12.88	1.95	0.413
E25.8	0.19	0.06	0.223
E25.9	1.01	0.27	0.482
E27.1	6.64	0.27	0.766
E27.2	1.86	0.12	0.389
E27.4	121.76	9.85	0.072
E34.8	1.09	−0.05	0.787
E55.0	11.05	−1.64	0.290
E70	0.00	.	.
E70.0	2.17	0.16	0.726
E70.1	2.29	0.08	0.601
E70.2	0.31	−0.22	0.076
E70.3	3.15	−0.39	0.345
E70.8	0.04	−0.04	0.182
E70.9	0.04	0.02	0.559
E71	0.23	0.12	0.559
E71.0	0.31	0.00	0.993
E71.1	2.02	0.18	0.274
E71.2	0.58	0.02	0.904
E71.3	4.00	0.33	0.437
E72	0.23	0.04	0.707
E72.0	1.79	−0.01	0.945
E72.1	1.75	0.26	0.450
E72.2	2.75	0.66	0.067
E72.3	0.31	0.00	0.964
E72.4	0.27	0.08	0.340
E72.5	0.51	−0.14	0.156
E72.8	0.50	0.09	0.379
E72.9	0.16	−0.02	0.539
E73.0	0.00	.	.
E73.1	0.04	0.02	0.559
E73.8	0.08	0.00	1.000
E73.9	0.23	−0.04	0.520
E74	0.12	0.08	0.183
E74.0	4.23	0.81	0.133
E74.1	0.00	.	.
E74.2	3.08	−0.50*	0.027
E74.3	0.16	0.04	0.698
E74.4	0.16	−0.06	0.314
E74.8	1.32	−0.24	0.076
E74.9	0.00	.	.
E75.0	0.19	0.00	0.985
E75.1	0.08	0.08	0.182
E75.2	5.16	1.14	0.161
E75.4	0.23	0.08	0.315
E75.5	2.13	0.46*	0.009
E76	0.00	.	.
E76.0	0.19	0.04	0.677
E76.1	1.13	−0.04	0.871
E76.2	0.62	−0.06	0.672
E76.3	0.43	0.21	0.071
E76.8	0.00	.	.
E76.9	0.00	.	.
E77	0.04	0.00	1.000
E77.0	0.27	0.12	0.547
E77.1	0.00	.	.
E77.9	0.00	.	.
E79.1	0.23	0.00	0.995
E80.2	1.28	−0.14	0.256
E83.0	16.61	−1.24	0.120
E83.3	5.98	0.86	0.282
E84	0.19	0.13	0.119
E84.0	0.23	−0.04	0.173
E84.1	0.08	−0.06	0.057
E84.9	0.19	−0.04	0.436
E85	1.98	0.12	0.685
E85.0	0.31	0.12	0.083
E85.2	0.19	0.15	0.161
E85.3	0.47	0.07	0.498
E85.4	5.00	0.77	0.084
E85.8	2.37	0.18	0.218
E85.9	16.11	1.49	0.112
F84.2	4.58	0.21	0.482
G10	5.74	1.14	0.065
G11	0.81	0.17	0.478
G11.0	0.66	−0.22	0.215
G11.1	10.17	1.43	0.266
G11.2	38.45	5.61	0.051
G11.3	0.66	−0.20	0.129
G11.4	10.45	0.60	0.494
G11.8	1.63	0.26*	0.030
G11.9	36.38	3.33	0.107
G12	0.97	0.19	0.189
G12.0	0.86	0.00	0.915
G12.1	9.38	2.32	0.208
G12.2	4.01	−0.77	0.367
G12.20	1.86	0.11	0.479
G12.21	55.69	7.32	0.287
G12.22	2.48	0.34	0.473
G12.23	5.83	0.09	0.905
G12.24	1.24	0.26	0.266
G12.8	3.03	−0.27	0.138
G12.9	6.06	0.46	0.662
G13	0.24	−0.20	0.162
G13.0	1.36	−0.30	0.242
G13.1	6.87	4.54*	0.013
G13.2	0.19	0.04	0.464
G13.8	2.88	−0.41	0.259
G20	2064.86	140.88*	0.029
G23.1	27.21	−0.79	0.850
G31.81	1.32	0.21	0.385
G35	45.14	−2.04	0.375
G40.4	24.10^c^	.	.
G40.40	52.65^c^	.	.
G40.41	33.90^c^	.	.
G41	8.74	0.18	0.683
G41.0	8.89	0.91	0.372
G41.1	1.32	0.42*	0.043
G41.2	13.85	2.08*	0.013
G41.8	15.68	2.22*	0.037
G41.9	124.11	15.76*	0.001
G51.2	0.19	−0.04	0.436
G56.4	36.98	−5.16*	0.015
G60.0	30.34	−2.22	0.166
G61	2.29	−0.17	0.575
G61.0	123.72	12.43*	0.021
G61.1	0.08	−0.02	0.629
G61.8	15.62	0.30	0.549
G61.9	8.50	0.73	0.408
G63.0	1.94	0.03	0.882
G70.0	137.88	14.27	0.150
G70.1	0.08	−0.02	0.637
G70.2	0.08	0.00	0.996
G71	2.72	0.31	0.548
G71.0	40.04	2.65	0.567
G71.1	33.29	0.95	0.389
G71.2	6.44	0.82	0.431
G71.3	12.33	−1.25	0.141
G71.8	0.81	0.25	0.288
G71.9	2.32	0.88	0.084
G90.8	2.68	0.06	0.839
G95.0	46.81	−1.70*	0.030
H35.31	1359.61	103.62	0.121
H35.51	163.56	1.17	0.869
H35.58	6.33	0.78*	0.016
I27.0	40.80	−3.17	0.204
I27.8	10.09	1.13	0.437
I42.0	521.39	44.36*	0.037
I42.1	42.31	4.15	0.139
I42.2	322.48	34.75	0.058
I42.3	1.09	0.11	0.489
I42.4	3.26	0.39	0.373
I42.5	7.10	0.99*	0.007
I67.5	261.73	0.38	0.888
I73.1	52.76	−5.97	0.080
I78.0	1.40	−0.39	0.203
I82.0	7.32	−1.02	0.104
J84.0	4.79	−0.86	0.135
J84.18	311.03	19.79*	0.006
K50	32.85	2.30	0.312
K50.0	51.57	6.73	0.262
K50.1	53.85	8.29	0.223
K50.8	35.33	6.61	0.126
K50.9	183.98	31.23	0.065
K51	52.66	−0.32	0.944
K51.0	67.17	9.06	0.218
K51.2	181.53	27.31*	0.019
K51.3	54.01	7.81	0.110
K51.4	0.19	0.02	0.732
K51.5	8.14	2.15	0.099
K51.8	63.29	8.08	0.163
K51.9	373.46	79.92	0.093
K74.3	68.32	6.72*	0.008
K75.4	104.26	7.90*	0.036
L10.0	13.94	1.14	0.246
L10.2	7.18	0.43	0.286
L12.0	50.83	7.27*	0.026
L12.1	0.46	0.15	0.082
L12.3	1.52	−0.14	0.555
M07.20	2.06	0.38*	0.010
M07.28	0.39	0.13	0.192
M08.0	4.58	0.68	0.575
M08.1	0.47	0.11	0.085
M08.2	0.27	0.04	0.717
M08.3	0.39	0.02	0.881
M30.0	7.08	−0.66	0.159
M30.1	9.75	0.66	0.313
M30.2	0.00	.	.
M31.0	2.72	0.35	0.266
M31.1	7.26	0.29	0.370
M31.2	0.00	.	.
M31.3	8.89	1.02	0.114
M31.4	26.14	1.38	0.378
M31.7	9.26	2.61*	0.009
M32.1	13.13	3.76	0.506
M32.10	4.76	2.21	0.286
M32.12	2.48	0.59	0.333
M32.13	46.43	18.53	0.341
M32.15	20.27	5.24	0.306
M32.19	46.45	16.72	0.320
M32.8	11.48	1.77	0.506
M32.9	282.52	72.81	0.355
M33	0.66	−0.02	0.858
M33.0	2.83	0.26	0.301
M33.1	22.36	2.58	0.198
M33.2	19.88	1.95	0.265
M33.9	6.25	0.80	0.485
M34.0	18.01	1.96	0.355
M34.1	3.61	0.35	0.434
M34.8	10.21	1.22	0.332
M34.9	57.97	7.94	0.379
M35.0	241.86	16.04*	0.036
M35.1	25.04	2.21	0.406
M35.2	265.39	−14.30	0.221
M35.3	56.31	7.45*	0.022
M35.4	1.13	−0.03	0.872
M35.5	0.08	0.02	0.646
M35.6	0.23	− 0.14	0.068
M35.7	0.47	0.06	0.621
M45	75.30	1.51	0.869
M61.1	0.51	−0.16	0.228
M88	0.23	0.02	0.858
M88.0	0.19	−0.04	0.436
M88.8	0.04	0.04	0.182
M88.9	0.16	0.00	0.992
M89.0	13.45	−3.51	0.131
M94.1	1.09	0.05	0.695
N25.1	61.63	−23.97*	0.006
P22.0	519.43	85.09*	0.001
Q03.1	4.86	0.01	0.966
Q04.3	2.60	0.08	0.732
Q04.6	8.16	−0.14	0.649
Q05	1.32	0.07	0.807
Q05.0	0.12	−0.10*	0.047
Q05.1	0.04	0.00	1.000
Q05.2	0.66	0.23	0.059
Q05.3	0.23	−0.10	0.273
Q05.4	0.39	0.04	0.814
Q05.5	0.12	0.00	0.984
Q05.6	0.16	−0.06	0.314
Q05.7	1.75	−0.11	0.477
Q05.8	1.01	0.03	0.825
Q05.9	41.94	3.03	0.163
Q06.2	0.51	−0.08	0.472
Q07.0	21.11	−0.27	0.718
Q20.0	1.75	− 0.07	0.809
Q20.1	22.26	0.36	0.548
Q20.2	0.62	0.15	0.312
Q20.4	19.02	5.58	0.127
Q21.8	3.22	0.17	0.681
Q22.0	17.12	1.84	0.086
Q22.6	1.32	0.32*	0.007
Q23	0.43	0.00	0.988
Q23.0	14.37	1.09*	0.039
Q23.1	96.01	16.07*	0.011
Q23.2	1.79	0.21	0.411
Q23.3	18.17	1.97	0.097
Q23.4	2.10	0.12	0.542
Q23.8	1.94	0.17	0.538
Q23.9	4.80	1.98	0.117
Q24.5	71.33	1.77	0.748
Q25.5	9.59	0.56	0.583
Q26.0	0.31	−0.04	0.790
Q26.1	6.28	1.79*	0.001
Q26.2	12.06	−0.85	0.213
Q26.3	8.14	1.58*	0.025
Q26.4	0.54	−0.02	0.726
Q26.5	0.62	−0.02	0.901
Q26.6	0.16	−0.02	0.751
Q38.3	0.08	−0.04	0.305
Q44.2	13.31	−1.30	0.059
Q64.1	0.66	0.25	0.180
Q75.1	2.53	−0.40	0.103
Q75.4	1.94	0.09	0.283
Q77	0.12	0.02	0.777
Q77.0	0.39	−0.02	0.691
Q77.2	0.12	−0.06	0.354
Q77.3	1.52	−0.09	0.487
Q77.4	8.98	−0.50	0.107
Q77.5	0.16	0.00	0.975
Q77.6	0.04	−0.04	0.182
Q77.7	2.45	−0.19	0.424
Q77.8	3.19	0.00	0.995
Q77.9	0.50	0.07	0.745
Q78.0	7.54	−0.18	0.793
Q78.1	4.90	0.13	0.695
Q78.2	1.79	−0.34	0.158
Q78.4	5.44	−0.38*	0.004
Q78.5	0.04	0.04	0.182
Q78.6	4.98	−0.18	0.846
Q79	0.20	−0.10	0.109
Q79.0	8.82	−0.03	0.955
Q79.1	5.94	0.51	0.354
Q79.2	3.76	0.50	0.194
Q79.3	1.83	0.13	0.640
Q79.4	0.23	0.04	0.644
Q79.5	0.43	0.10	0.288
Q79.6	1.13	−0.14	0.616
Q79.8	7.07	0.19	0.609
Q79.9	5.17	−0.23	0.386
Q81.1	0.04	−0.04	0.182
Q81.2	1.05	−0.20	0.397
Q85.0	117.58	−12.67	0.080
Q85.1	17.35	−1.52*	0.050
Q85.8	14.18	0.50	0.684
Q86.0	0.04	0.04	0.182
Q87.0	8.67	−0.69	0.193
Q87.1	19.00	0.09	0.912
Q87.2	8.84	5.42*	0.050
Q87.3	2.06	−0.07	0.809
Q87.4	29.03	−0.11	0.910
Q90	7.10	0.89	0.293
Q90.0	5.32	0.59	0.347
Q90.1	0.23	0.17	0.058
Q90.2	0.23	0.11	0.167
Q90.9	48.67	6.03	0.278
Q91	0.00	.	.
Q91.0	0.12	−0.08	0.181
Q91.1	0.00	.	.
Q91.2	0.04	0.00	1.000
Q91.3	0.70	0.05	0.583
Q91.4	0.04	0.04	0.182
Q91.5	0.08	0.06	0.058
Q91.7	0.15	0.06	0.324
Q93.4	2.14	0.03	0.946
Q93.5	10.64	1.05	0.307
Q96	3.15	0.06	0.896
Q96.0	2.13	0.30	0.533
Q96.1	0.43	0.17	0.293
Q96.2	0.04	0.02	0.559
Q96.3	1.28	0.26	0.265
Q96.4	0.54	0.21	0.105
Q96.8	3.92	0.16	0.553
Q96.9	32.59	5.01	0.216
Q98.0	11.85	0.64	0.586
Q98.1	0.62	0.09*	0.017
Q98.2	0.35	0.10	0.288
Q98.4	4.19	0.23	0.632
Q99.2	1.24	0.44	0.197

Abbreviation: *KCD* Korean Standard Classification of Diseases

^a^Average of annual cumulative incidence per 10,000,000 of rare diseases from 2011 to 2015 according to the KCD-7 code

^b^linear trend of annual cumulative incidence per 10,000,000 per year

^c^Average of annual cumulative incidence per 10,000,000 of rare diseases from 2014 to 2015 according to the KCD-7 code

*β was statistically significant (*p* value < 0.05)

The trends in annual cumulative incidence for 43 rare diseases were statistically significant (*p*-value < 0.05). Among them, 34 rare diseases including sarcoidosis (D86.0, D86.1, D86.2), Parkinson’s disease (G20), Guillain-Barré syndrome (G61.0), idiopathic pulmonary fibrosis (J84.18), primary biliary cirrhosis (K74.3), autoimmune hepatitis (K75.4), bullous pemphigoid (L12.0), microscopic polyangiitis (M31.7) and Sjogren’s syndrome (M35.0) showed an increasing trend and 9 rare diseases including neutropenia (D70), nephrogenic diabetes insipidus (N25.1) and tuberous sclerosis (Q85.1) showed a decreasing trend. The rare diseases, including those with the top 10 highest average annual cumulative incidences and for which increment trends per year were statistically significant were Parkinson’s disease (G20; β = 140.88; *p*-value = 0.029), dilated cardiomyopathy, congestive cardiomyopathy (I42.0; β = 44.36; *p*-value = 0.037), respiratory distress syndrome of the newborn (P22.0; β = 85.09; *p*-value = 0.001), and idiopathic pulmonary fibrosis (J84.18; β = 19.79; *p*-value = 0.006).

## Discussion

This study determined the annual cumulative incidence of rare diseases in Korea using nationwide data obtained from the administrative register for the co-payment assistance policy for rare and incurable diseases in the Korean NHIS from 2011 to 2015. Our results showed that the number of rare diseases showing an increasing trend in average annual cumulative incidence was higher than the number of diseases showing a decreasing trend in average annual cumulative incidence (34 [8.19%] vs. 4 [0.96%]).

The rare disease with the highest average annual cumulative incidences and for which the increment trends per year were statistically significant was Parkinson’s disease. Parkinson’s disease was the most common rare disease (average annual cumulative incidence: 2066.43/10,000,000) and its annual cumulative incidence increased from 2011 to 2015 in our study. The annual cumulative incidence of Parkinson’s disease per 10,000,000 was 1884.36 in 2011, 1882.58 in 2012, 1945.05 in 2013, 2164.53 in 2014 and 2447.80 in 2015 (see Additional file 2). Parkinson’s disease is a representative age-related neurodegenerative disease [6]. Several studies reported that older age was major risk factor for an increased risk of developing Parkinson’s disease [25]. South Korea is undergoing unprecedented and rapid population aging and life expectancy at birth has increased steadily from 80.62 in 2011 to 82.06 in 2015 [26]. Increased longevity might increase the incidence of Parkinson’s disease in South Korea. Considering the high burden and low quality of life observed with Parkinson’s disease, urgent attention should be directed towards developing national support systems to alleviate the burden on patients’ families [27, 28].

The number patients with sarcoidosis (D86, D86.0, D86.1, D86.2, D86.3, D86.8, D86.9) has also increased over the time. The annual cumulative incidence increased from 70.91 per 10,000,000 in 2011 to 101.86 per 10,000,000 in 2015 (see Additional file 2). Sarcoidosis is a multisystem disorder of which the aetiology is unknown. The case-control study of 706 newly diagnosed patients conducted by National Institutes of Health found positive associations between sarcoidosis and exposures including insecticides, agricultural employment and microbial bioaerosols [29]. However, these exposures have decreased in Korea due to the shift of the economy from agriculture to the manufacturing and service sector. The increasing trend in the annual cumulative incidence of sarcoidosis might be explained by the increased detection rate of sarcoidosis due to the increased diagnostic techniques and frequent regular health check-ups [30–32]. The overall participation rate in primary health examinations, including the chest X-rays, has increased from 56% in 2006 to 72% in 2013 [32]. The number of patients using computed tomography and magnetic resonance imaging, which are diagnostic tools for sarcoidosis, have also increased steadily from 4,118,434 and 631,305 in 2012 to 5,139,149 and 805,831 in 2015, respectively [33].

Upon comparison of the cumulative incidence of rare diseases in our study to the estimated incidence reported by the Orphanet [20], we observed a significant difference in the incidence of Moyamoya disease (I67.5; our result: 261.73/10,000,000 vs Orphanet: 3.5/10,000,000). A previous systematic review revealed that the incidence of definite Moyamoya disease in Asia (Japan, Hokkaido: 84 per 10,00,000; China: 41 per 10,00,000) was higher than in the USA (Iowa, 4 per 10,000,000) [34]. Although Asian countries have a predilection for developing Moyamoya disease, the cumulative incidence of Moyamoya disease in South Korea was higher than in other Asian countries. This high incidence among South Koreans has also been supported by the findings from other studies which have used the medical claim data of NHIS (average annual cumulative incidence 2005–2013: 270/10,000,000) [35]. The high incidence of Moyamoya disease in East Asia might be partly explained by genetic effects of developing the rare disease [36]. A previous study showed a strong association between the p.R4810K mutation in RNF213 [24], which has a relatively high prevalence among East Asians and is a risk factor for the development of Moyamoya disease [37].

To the best of our knowledge, this is the first study to estimate the cumulative incidence of an entire spectrum of rare diseases in Korea by using data from a nationwide administrative database in the register of rare diseases. However, our results should be interpreted within the context of the study’s limitations. First, only patients with rare diseases who were supported by the government were included in our study. Considering the complexity of the diagnosis of rare diseases, there is a possibility that many patients with rare diseases did not receive a definite diagnosis by the physician, which is a prerequisite for national support for rare diseases. Furthermore, the method of grouping patients with rare diseases according to the ICD codes could overestimate the actual incidence of rare diseases when the specificity of the ICD coding was not validated [38]. Therefore, our estimated annual cumulative incidence might not reflect the actual incidence of rare diseases. However, our results do supplement the disadvantages observed in other studies which have used the ICD codes in medical claim data leading to the ambiguity of the diagnosis of rare diseases. Our study included patients who met the diagnostic criteria on the basis of imaging studies, biochemistry, immunology, smear, culture test, histological examination and clinical diagnosis for benefiting from the national support. Second, our study could not include all rare diseases for which the prevalence was less than 20,000 patients in South Korea. Particularly, our result could not show the annual cumulative incidence of almost all cases of cancer because the medical expenses were covered by other co-payment assistance policies established by the NHIS. Third, owing to the available options for KCD codes on the application form of the register of the co-payment assistance policy for rare and incurable diseases, multiple diseases with similar characteristics were included under one KCD code. We could not separately analyse each rare disease included under one KCD code using these administrative data. Fourth, our study used the year of definite diagnosis by the physician in the application form as the year of disease occurrence. Because the definite diagnosis of a disease is usually confirmed after the disease progresses enough to affect the quality of life of patients [11], the year of disease onset in our study might not represent the actual incident date of the rare disease. Fifth, the total number of rare diseases increased abruptly from 2013 to 2014 after the Ministry of Health and Welfare of Korea adopted the agenda for expanding the health coverage for rare and incurable diseases. The number of patients enrolled in the register of the co-payment assistance policy was not constant and varied based on government policy, which might have therefore disrupted the calculation of the actual incidence of the rare diseases. For yearly comparisons of the incidence of each rare disease, the annual cumulative incidence of each rare disease is shown in Additional file 2. Sixth, no cases of 18 (4.34%) KCD code diseases (D56, D81, D82, D83, D84.0, E70, E73.0, E74.1, E74.9, E76, E76.8, E76.9, E77.1, E77.9, M30.2, M31.2, Q91, Q91.1) were reported during the study period. Given that the NHIS determined the target disease of the co-payment policy before 2011, it is possible that there were no cases of registrants with these KCD codes for 2011–2015. Seventh, in order to calculate the incidence of rare disease, the denominator should be limited to the “population at risk” for developing rare disease. However, we set the denominator as the number of residents with health insurance coverage in each year. Additional studies are needed to better understand the epidemiology of each rare disease by using administrative records of rare disease registries in Korea.

## Conclusion

We analysed the average annual cumulative incidence of rare diseases and the trends in the annual cumulative incidence per year from 2011 to 2015 in Korea by using data from the nationwide administrative database complied by the Korean NHIS. The number of rare diseases showing an increasing trend in annual cumulative incidence was higher than the number of diseases showing a decreasing trend in annual cumulative incidence. Although we calculated the annual cumulative incidence of all the rare diseases documented in Korea, it is difficult to evaluate the precise epidemiology of these rare diseases considering that the definition and diagnosis varies by country and considering the difficulty with detecting valid cases. Further detection strategies are needed to establish the incidence of each rare disease, considering the importance of establishing a health policy based on the actual incidence of targeted diseases.

## Additional files

Additional file 1: **Table S1.** Targeted rare diseases included in the co-payment assistance policy established by the NHIS according to KCD codes in Korea. Description of data: Additional file 1 includes the full list of targeted rare diseases covered by the co-payment assistance policy for rare and incurable diseases in South Korea. Registered patients with rare diseases make out-of-pocket payments that comprise about 10% of the total cost of medical treatment, which is normally 20%–60% of the total cost of treatment. (DOCX 48 kb)

Additional file 2: **Table S2.** Annual cumulative incidence per 10,000,000 insured population for rare diseases in the register of the co-payment assistance policy according to KCD codes from 2011–2015. Description of data: Additional file 2 includes the annual cumulative incidence per 10,000,000 for each targeted rare disease from 2011–2015. Annual cumulative incidence per 10,000,000 was calculated as the total number of newly enrolled patients with the KCD-7 code in the register of the co-payment assistance policy for rare and incurable diseases during a calendar year, divided by the number of residents with health insurance coverage in each year. (DOCX 80 kb)

## Abbreviations

ICD: International Classification of Diseases
KCD: Korean standard classification of diseases
NHI: National Health Insurance
NHIS: National Health Insurance Service
