# Supplementary material for: The cumulative incidence and trends of rare diseases in South Korea: a nationwide study of the administrative data from the National Health Insurance Service database from 2011–2015
Source: Orphanet J Rare Dis. 2019 Feb 18;14:49. doi: 10.1186/s13023-019-1032-6 (PMC6379926; doi:10.1186/s13023-019-1032-6)
Supplement: Supplementary file 1 — Table S1. Targeted rare diseases included in the co-payment assistance policy established by the NHIS according to KCD codes in Korea. Description of data: Additional file 1 includes the full list of targeted rare diseases covered by the co-payment assistance policy for rare and incurable diseases in South Korea. Registered patients with rare diseases make out-of-pocket payments that comprise about 10% of the total cost of medical treatment, which is normally 20%–60% of the total cost of treatment. (DOCX 48 kb) [file 13023_2019_1032_MOESM1_ESM.docx]

Table S1. Targeted rare diseases included in the co-payment assistance policy established by the NHIS according to KCD codes in Korea

| KCD | Rare and incurable disease |
| --- | --- |
| A81 | Atypical virus infections of central nervous system |
| A81.0 | Creutzfeldt-Jakob disease Subacute spongiform encephalopathy |
| A81.1 | Subacute sclerosing panencephalitis Dawson’s inclusion body encephalitis Van Bogaert’s sclerosing leukoencephalopathy |
| A81.2 | Progressive multifocal leukoencephalopathy Multifocal leukoencephalopathy NOS |
| A81.8 | Other atypical virus infections of central nervous system Kuru |
| A81.9 | Atypical virus infection of central nervous system, unspecified Prion disease of central nervous system NOS |
| B45 | Cryptococcosis |
| B45.0 | Pulmonary cryptococcosis |
| B45.1 | Cerebral cryptococcosis Cryptococcal meningitis Cryptococcal meningocerebritis |
| B45.2 | Cutaneous cryptococcosis |
| B45.3 | Osseous cryptococcosis |
| B45.7 | Disseminated cryptococcosis Generalized cryptococcosis |
| B45.8 | Other forms of cryptococcosis |
| B45.9 | Cryptococcosis, unspecified |
| D35.2 | Benign neoplasm of pituitary gland |
| D55.0 | Anaemia due to glucose-6- phosphate dehydrogenase [G6PD] deficiency Favism G6PD deficiency anaemia |
| D55.2 | Anaemia due to disorders of glycolytic enzymes Haemolytic nonspherocytic (hereditary), type Ⅱ anaemia Hexokinase deficiency anaemia Pyruvate kinase[PK] deficiency anaemia Triose-phosphate isomerase deficiency anaemia |
| D56 | Thalassaemia |
| D56.0 | Alpha thalassaemia |
| D56.1 | Beta thalassaemia Cooley’s anaemia Severe beta thalassaemia Intermedia thalassaemia Major thalassaemia |
| D56.3 | Thalassaemia trait |
| D56.4 | Hereditary persistence of fetal haemoglobin [HPFH] |
| D56.8 | Other thalassaemias |
| D56.9 | Thalassaemia, unspecified Mediterranean anaemia (with other haemoglobinopathy) Thalassaemia(minor, mixed, with other haemoglobinopathy) |
| D59.5 | Paroxysmal nocturnal haemoglobinuria [Marchiafava-Micheli] |
| D60 | Acquired pure red cell aplasia [erythroblastopenia] |
| D60.0 | Chronic acquired pure red cell aplasia |
| D60.1 | Transient acquired pure red cell aplasia |
| D60.8 | Other acquired pure red cell aplasias |
| D60.9 | Acquired pure red cell aplasia, unspecified |
| D61.0 | Constitutional aplastic anaemia Aplasia, (pure) red cell (of) congenital Aplasia, (pure) red cell (of) infants Aplasia, (pure) red cell (of) primary Blackfan-Diamond syndrome Familial hypoplastic anaemia Fanconi’s anaemia Pancytopenia with malformations |
| D61.2 | Aplastic anaemia due to other external agents |
| D61.3 | Idiopathic aplastic anaemia |
| D61.8 | Other specified aplastic anaemias |
| D61.9 | Aplastic anaemia, unspecified Hypoplastic anaemia NOS Medullary hypoplasia Panmyelophthisis |
| D64.4 | Congenital dyserythropoietic anaemia Dyshaematopoietic anaemia(congenital) |
| D69.1 | Qualitative platelet defects Bernard-Soulier[giant platelet] syndrome Glanzmann’s disease Grey platelet syndrome Thromboasthenia (haemorrhagic)(hereditary) Thrombocytopathy |
| D69.30 | Evans’ syndrome |
| D69.6 | Thrombocytopenia, unspecified |
| D70 | Agranulocytosis Agranulocytic angina Infantile genetic agranulocytosis Kostmann’s disease Neutropenia NOS Congenital neutropenia Cyclic neutropenia Periodic neutropenia Splenin (primary) neutropenia Toxic neutropenia Neutropenic splenomegaly |
| D71 | Functional disorders of polymorphonuclear neutrophils Cell membrane receptor complex [CR3] defect Chronic (childhood) granulomatous disease Congenital dysphagocytosis Progressive septic granulomatosis |
| D76.1 | Haemophagocytic lymphohistiocytosis Familial haemophagocytic reticulosis Histiocytoses of mononuclear phagocytes |
| D76.3 | Other histiocytosis syndromes Reticulohistiocytoma(giant-cell) Sinus histiocytosis with massive lymph- adenopathy Xanthogranuloma |
| D80 | Immunodeficiency with predominantly antibody defects |
| D80.0 | Hereditary hypogammaglobulinaemia Autosomal recessive agammaglobulinaemia (Swiss type) X-linked agammaglobulinaemia [Bruton] (with growth hormone deficiency) |
| D80.1 | Nonfamilial hypogammaglobulinaemia Agammaglobulinaemia with immunoglobulin-bearing B-lymphocytes Common variable agammaglobulinaemia [CVAgamma] Hypogammaglobulinaemia NOS |
| D80.2 | Selective deficiency of immunoglobulin A[IgA] |
| D80.3 | Selective deficiency of immunoglobulin G [IgG] subclasses |
| D80.4 | Selective deficiency of immunoglobulin M[IgM] |
| D80.5 | Immunodeficiency with increased immunoglobulin M[IgM] |
| D80.6 | Antibody deficiency with near-normal immunoglobulins or with hyperimmunoglobulinaemia |
| D80.7 | Transient hypogammaglobulinaemia of infancy |
| D80.8 | Other immunodeficiencies with predominantly antibody defects Kappa light chain deficiency |
| D80.9 | Immunodeficiency with predominantly antibody defects, unspecified |
| D81 | Combined immunodeficiencies |
| D81.1 | Severe combined immunodeficiency [SCID] with low T-and B-cell numbers |
| D81.2 | Severe combined immunodeficiency [SCID] with low or normal B-cell numbers |
| D81.8 | Other combined immunodeficiencies Biotin-dependent carboxylase deficiency |
| D81.9 | Combined immunodeficiency, unspecified Severe combined immunodeficiency disorder [SCID]) NOS |
| D82 | Immunodeficiency associated with other major defects |
| D82.0 | Wiskott-Aldrich syndrome Immunodeficiency with thrombocy- topenia and eczema |
| D82.1 | Di George’s syndrome Pharyngeal pouch syndrome Thymic alymphoplasia Thymic aplasia or hypoplasia with immunodeficiency |
| D82.3 | Immunodeficiency following hereditary defective response to Epstein-Barr virus X-linked lymphoproliferative disease |
| D82.4 | Hyperimmunoglobulin E [IgE] syndrome |
| D82.8 | Immunodeficiency associated with other specified major defects |
| D83 | Common variable immunodeficiency |
| D83.0 | Common variable immunodeficiency with predominant abnormalities of B-cell numbers and function |
| D83.9 | Common variable immunodeficiency, unspecified |
| D84 | Other immunodeficiencies |
| D84.0 | Lymphocyte function antigen-1[LFA-1] defect |
| D84.1 | Defects in the complement system C1 esterase inhibitor[C1-INH] deficiency |
| D84.8 | Other specified immunodeficiencies |
| D84.9 | Immunodeficiency, unspecified |
| D86 | Sarcoidosis |
| D86.0 | Sarcoidosis of lung |
| D86.1 | Sarcoidosis of lymph nodes |
| D86.2 | Sarcoidosis of lung with sarcoidosis of lymph nodes |
| D86.3 | Sarcoidosis of skin |
| D86.8 | Sarcoidosis of other and combined sites Iridocyclitis in sarcoidosis Multiple cranial nerve palsies in sarcoidosis Sarcoid arthropathy Sarcoid myocarditis Sarcoid myositis Uveoparotid fever[Heerfordt] |
| D86.9 | Sarcoidosis, unspecified |
| E22.0 | Acromegaly and pituitary gigantism Arthropathy associated with acromegaly Overproduction of growth hormone |
| E23.0 | Kallmann’s syndrome Sheehan’s syndrome |
| E24.0 | Pituitary-dependent Cushing’s disease Overproduction of pituitary ACTH Pituitary-dependent hyperadrenocorticism |
| E24.1 | Nelson’s syndrome |
| E24.3 | Ectopic ACTH syndrome |
| E25 | Adrenogenital disorders |
| E25.0 | Congenital adrenogenital disorders associated with enzyme deficiency Congenital adrenal hyperplasia 21-Hydroxylase deficiency Salt-losing congenital adrenal hyperplasia |
| E25.8 | Other adrenogenital disorders Idiopathic adrenogenital disorder |
| E25.9 | Adrenogenital disorder, unspecified Adrenogenital syndrome NOS |
| E27.1 | Primary adrenocortical insufficiency Addison’s disease Autoimmune adrenalitis |
| E27.2 | Addisonian crisis Adrenal crisis Adrenocortical crisis |
| E27.4 | Other and unspecified adrenocortical insufficiency Adrenal haemorrhage Adrenal infarction Adrenocortical insufficiency NOS Hypoaldosteronism |
| E34.8 | Other specified endocrine disorders Pineal gland dysfunction Progeria |
| E55.0 | Rickets, active Infantile osteomalacia Juvenile osteomalacia |
| E70 | Disorders of aromatic amino-acid metabolism |
| E70.0 | Classical phenylketonuria |
| E70.1 | Other hyperphenylalaninaemias |
| E70.2 | Disorders of tyrosine metabolism Alkaptonuria Hypertyrosinaemia Ochronosis Tyrosinaemia Tyrosinosis |
| E70.3 | Albinism Oculocutaneous albinism Ocular albinism Chediack(-Steinbrinck)-Higashi syndrome Cross syndrome Hermansky-Pudlak syndrome |
| E70.8 | Other disorders of aromatic amino-acid metabolism Disorder of histidine metabolism Disorder of tryptophan metabolism |
| E70.9 | Disorder of aromatic amino-acid metabolism, unspecified |
| E71 | Disorders of branched-chain amino-acid metabolism and fatty-acid metabolism |
| E71.0 | Maple-syrup-urine disease |
| E71.1 | Other disorders of branched-chain amino-acid metabolism Hyperleucine-isoleucinaemia Hypervalinaemia Methylmalonic acidaemia Isovaleric acidaemia Propionic acidaemia |
| E71.2 | Disorder of branched-chain amino-acid metabolism, unspecified |
| E71.3 | Disorders of fatty-acid metabolism Adrenoleukodystrophy[Addison-Schilder] Muscle carnitine palmityltransferase deficiency |
| E72 | Other disorders of amino-acid metabolism |
| E72.0 | Disorders of amino-acid transport Cystinosis Cystinuria Fanconi(-de Toni)(-Debré)syndrome Hartnup’s disease Lowe’s syndrome |
| E72.1 | Disorders of sulfur-bearing amino-acid metabolism Cystathioninuria Homocystinuria Methioninaemia Sulfite oxidase deficiency |
| E72.2 | Disorders of urea cycle metabolism Argininaemia Argininosuccinic aciduria Citrullinaemia Hyperammonaemia |
| E72.3 | Disorders of lysine and hydroxylysine metabolism Glutaric aciduria Hydroxylysinaemia Hyperlysinaemia |
| E72.4 | Disorders of ornithine metabolism Ornithinaemia (typesⅠ, Ⅱ) |
| E72.5 | Disorders of glycine metabolism Hyperhydroxyprolinaemia Hyperprolinaemia (typesⅠ, Ⅱ) Non-ketotic hyperglycinaemia Sarcosinaemia |
| E72.8 | Other specified disorders of amino-acid metabolism Disorders of β-amino-acid metabolism Disorders of γ-glutamyl cycle |
| E72.9 | Disorder of amino-acid metabolism, unspecified |
| E73.0 | Congenital lactase deficiency |
| E73.1 | Secondary lactase deficiency |
| E73.8 | Other lactose intolerance |
| E73.9 | Lactose intolerance, unspecified |
| E74 | Other disorders of carbohydrate metabolism |
| E74.0 | Glycogen storage disease Cardiac glycogenosis Andersen’s disease Cori’s disease Forbes’ disease Hers’ disease McArdle’s disease Pompe’s disease Tarui’s disease von Gierke’s disease Liver phosphorylase deficiency |
| E74.1 | Disorders of fructose metabolism Essential fructosuria Fructose-1, 6-diphosphatase deficiency Hereditary fructose intolerance |
| E74.2 | Disorders of galactose metabolism Galactokinase deficiency Galactosaemia |
| E74.3 | Other disorders of intestinal carbohydrate absorption Glucose-galactose malabsorption Sucrase deficiency |
| E74.4 | Disorders of pyruvate metabolism and gluco- neogenesis Deficiency of phosphoenol- pyruvate carboxykinase Deficiency of carboxylase pyruvate Deficiency of dehydrogenase pyruvate |
| E74.8 | Other specified disorders of carbohydrate metabolism Essential pentosuria Oxalosis Oxaluria Renal glycosuria |
| E74.9 | Disorder of carbohydrate metabolism, unspecified |
| E75.0 | GM₂-gangliosidosis Sandhoff’s disease Tay-Sachs’ disease GM₂gangliosidosis NOS Adult GM₂gangliosidosis Juvenile GM₂gangliosidosis |
| E75.1 | Other gangliosidosis Gangliosidosis NOS GM₁-gangliosidosis GM₃-gangliosidosis Mucolipidosis Ⅳ |
| E75.2 | Other sphingolipidosis Fabry’s(-Anderson) disease Gaucher’s disease Krabbe’s disease Niemann-Pick’s disease Farber’s syndrome Metachromatic leukodystrophy Sulfatase deficiency |
| E75.4 | Neuronal ceroid lipofuscinosis Batten’s disease Jansky-Bielschowsky’s disease Kufs’ disease Spielmeyer-Vogt’s disease |
| E75.5 | Other lipid storage disorders Cerebrotendinous cholesterosis [van Bogaert-Scherer-Epstein] Wolman’s disease |
| E76 | Disorders of glycosaminoglycan meta- bolism |
| E76.0 | Mucopolysaccharidosis, type Ⅰ Hurler syndrome Hurler-Scheie syndrome Scheie syndrome |
| E76.1 | Mucopolysaccharidosis, type Ⅱ Hunter’s syndrome |
| E76.2 | Other mucopolysaccharidoses β-Glucuronidase deficiency Mucopolysaccharidosis, types Ⅲ, Ⅳ, Ⅵ, Ⅶ Maroteaux-Lamy (mild)(severe) syndrome Morquio(-like)(classic) syndrome Sanfilippo (types B)(type C)(type D) syndrome |
| E76.3 | Mucopolysaccharidosis, unspecified |
| E76.8 | Other disorders of glucosaminoglycan metabolism |
| E76.9 | Disorders of glucosaminoglycan metabolism, unspecified |
| E77 | Disorders of glycoprotein metabolism |
| E77.0 | Defects in post-translational modification of lysosomal enzymes Mucolipidosis Ⅱ[Ⅰ-cell disease] Mucolipidosis Ⅲ [Pseudo-Hurler polydystrophy] |
| E77.1 | Defects in glycoprotein degradation Aspartylglucosaminuria Fucosidosis Mannosidosis Sialidosis[mucolipidosisⅠ] |
| E77.9 | Disorder of glycoprotein metabolism, unspecified |
| E79.1 | Lesch-Nyhan syndrome |
| E80.2 | Other porphyria Hereditary coproporphyria Selective deficiency of immunoglobulin A[IgA] Acute intermittent(hepatic) porphyria |
| E83.0 | Disorders of copper metabolism Menkes (kinky hair)(steely hair) disease Wilson’s disease |
| E83.3 | Disorders of phosphorus metabolism and phosphatases Acid phosphatase deficiency Familial hypophosphataemia Hypophosphatasia Vitamin-D-resistant osteomalacia Vitamin-D-resistant rickets |
| E84 | Cystic fibrosis |
| E84.0 | Cystic fibrosis with pulmonary manifestations |
| E84.1 | Cystic fibrosis with intestinal manifestations Meconium ileus in cystic fibrosis |
| E84.9 | Cystic fibrosis, unspecified |
| E85 | Amyloidosis |
| E85.0 | Non-neuropathic heredofamilial amyloidosis Familial Mediterranean fever Hereditary amyloid nephropathy |
| E85.2 | Heredofamilial amyloidosis, unspecified |
| E85.3 | Secondary systemic amyloidosis Haemodialysis-associated amyloidosis |
| E85.4 | Organ-limited amyloidosis Localized amyloidosis |
| E85.8 | Other amyloidosis |
| E85.9 | Amyloidosis, unspecified |
| F84.2 | Rett’s syndrome |
| G10 | Huntington’s disease Huntington’s chorea |
| G11 | Hereditary ataxia |
| G11.0 | Congenital nonprogressive ataxia |
| G11.1 | Early-onset cerebellar ataxia Early-onset cerebellar ataxia with essential tremor Early-onset cerebellar ataxia with myoclonus[Hunt’s ataxia] Early-onset cerebellar ataxia with retained tendon reflexes Friedreich’s ataxia(autosomal recessive) X-linked recessive spinocerebellar ataxia |
| G11.2 | Late-onset cerebellar ataxia |
| G11.3 | Cerebellar ataxia with defective DNA repair Ataxia telangiectasia[Louis-bar] |
| G11.4 | Hereditary spastic paraplegia |
| G11.8 | Other hereditary ataxias |
| G11.9 | Hereditary ataxia, unspecified Hereditary cerebellar aataxia NOS Hereditary cerebellar degeneration Hereditary cerebellar disease Hereditary cerebellar syndrome |
| G12 | Spinal muscular atrophy and related syndromes |
| G12.0 | Infantile spinal muscular atrophy, type Ⅰ[Werdnig-Hoffman] |
| G12.1 | Other inherited spinal muscular atrophy Progressive bulbar palsy of childhood [Fazio-Londe] Adult form spinal muscular atrophy Childhood form, type Ⅱ spinal muscular atrophy Distal spinal muscular atrophy Juvenile form, typeⅢ [Kugelberg-Welander] spinal muscular atrophy Scapuloperoneal form spinal muscular atrophy |
| G12.2 | Motor neuron disease |
| G12.20 | Familial amyotrophic lateral sclerosis |
| G12.21 | Sporadic amyotrophic lateral sclerosis |
| G12.22 | Primary lateral sclerosis |
| G12.23 | Progressive bulbar palsy |
| G12.24 | Progressive muscular atrophy |
| G12.8 | Other spinal muscular atrophies and related syndromes |
| G12.9 | Spinal muscular atrophy, unspecified |
| G13 | Systemic atrophies primarily affecting central nervous system in diseases classified elsewhere |
| G13.0 | Paraneoplastic neuromyopathy and neuropathy Carcinomatous neuromyopathy Sensorial paraneopastic neuropathy [Denny Brown] |
| G13.1 | Other systemic atrophy primarily affecting central nervous system in neoplastic disease Paraneoplastic limbic encephalopathy |
| G13.2 | Systemic atrophy primarily affecting central nervous system in myxoedema |
| G13.8 | Systemic atrophy primarily affecting central nervous system in other diseases classified elsewhere |
| G20 | Parkinson’s disease Hemiparkinsonism Paralysis agitans Parkinsonism or Parkinson’s disease NOS Idiopathic Parkinsonism or Parkinson’s disease  Primary Parkinsonism or Parkinson’s disease |
| G23.1 | Progressive supranuclear ophthalmoplegia [Steele-Richardson-Olszewski] |
| G31.81 | Subacute necrotizing encephalopathy[Leigh] |
| G35 | Multiple sclerosis Multiple sclerosis (of) NOS Multiple sclerosis (of) brain stem Multiple sclerosis (of) cord Multiple sclerosis (of) disseminated Multiple sclerosis (of) generalized |
| G40.4 | Lennox-Gastaut syndrome |
| G40.40 | Lennox-Gastaut syndrome without intractable epilepsy |
| G40.41 | Lennox-Gastaut syndrome with intractable epilepsy |
| G41 | Status epilepticus |
| G41.0 | Grand mal status epilepticus Tonic-clonic status epilepticus |
| G41.1 | Petit mal status epilepticus Epileptic absence statu |
| G41.2 | Complex partial status epilepticus |
| G41.8 | Other status epilepticus |
| G41.9 | Status epilepticus, unspecified |
| G51.2 | Melkersson-Rosenthal syndrome |
| G56.4 | Complex regional pain syndrome typeⅡ Causalgia |
| G60.0 | Hereditary motor and sensory neuropathy Charcot-Marie-Tooth disease Déjerine-Sottas disease Hereditary motor and sensory neuropathy, types I-IV Hypertrophic neuropathy of infancy Peroneal muscular atrophy(axonal type, hypertrophic type) Roussy-Lévy syndrome |
| G61 | Inflammatory polyneuropathy |
| G61.0 | Guillain-Barré syndrome Acute (post-) infective polyneuritis |
| G61.1 | Serum neuropathy |
| G61.8 | Other inflammatory polyneuropathies |
| G61.9 | Inflammatory polyneuropathy, unspecified |
| G63.0 | Polyneuropathy ininfectious and parasitic diseases classified elsewhere Polyneuropathy (in) diphtheria Polyneuropathy (in) infectious mononucleosis Polyneuropathy (in) leprosy Polyneuropathy (in) lyme disease Polyneuropathy (in) mumps Polyneuropathy (in) postherpetic Polyneuropathy (in) syphilis, late Polyneuropathy (in) congenital syphilis, late Polyneuropathy (in) tuberculous |
| G70.0 | Myasthenia gravis |
| G70.1 | Toxic myoneural disorders |
| G70.2 | Congenital and developmental myasthenia |
| G71 | Primary disorders of muscles |
| G71.0 | Muscular dystrophy Autosomal recessive, childhood type, resembling Duchenne or Becker muscular dystrophy Benign [Becker] muscular dystrophy Benign scapuloperoneal with early contractures [Emery-Dreifuss] muscular dystrophy Distal muscular dystrophy Facioscapulohumeral muscular dystrophy Limb-girdle muscular dystrophy Ocular muscular dystrophy Oculopharyngeal muscular dystrophy Scapuloperoneal muscular dystrophy Severe[Duchenne] muscular dystrophy |
| G71.1 | Myotonic disorders Dystrophia myotonica[Steinert] Chondrodystrophic myotonia Drug-induced myotonia Symptomatic myotonia Myotonia congenita NOS Dominant[Thomsen] myotonia congenita  Recessive[Becker] myotonia congenita Neuromyotonia[Isaacs] Paramyotonia congenita Pseudomyotonia |
| G71.2 | Congenital myopathies Congenital muscular dystrophy NOS Congenital muscular dystrophy with specific morphological abnormalities of the muscle fibre Central core disease Minicore disease Multicore disease Fibre-type disproportion Myotubular (centronuclear) myopathy Nemaline myopathy |
| G71.3 | Mitochondrial myopathy, NEC |
| G71.8 | Other primary disorders of muscles |
| G71.9 | Primary disorder of muscle, unspecified Hereditary myopathy NOS |
| G90.8 | Other disorders of autonomic nervous system |
| G95.0 | Syringomyelia and syringobulbia |
| H35.31 | Senile macular degeneration (exudative) |
| H35.51 | Retinitis pigmentosa |
| H35.58 | Stargardt’s disease |
| I27.0 | Primary pulmonary hypertension |
| I27.8 | Eisenmenger’s complex Eisenmenger’s syndrome |
| I42.0 | Dilated cardiomyopathy Congestive cardiomyopathy |
| I42.1 | Obstructive hypertrophic cardiomyopathy Hypertrophic subaortic stenosis |
| I42.2 | Other hypertrophic cardiomyopathy Nonobstructive hypertrophic cardiomyopathy |
| I42.3 | Endomyocardial(eosinophilic) disease Endomyocardial(tropical) fibrosis Loffler’s endocarditis |
| I42.4 | Endocardial fibroelastosis Congenital cardiomyopathy |
| I42.5 | Other restrictive cardiomyopathy Constrictive cardiomyopathy NOS |
| I67.5 | Moyamoya disease |
| I73.1 | Thromboangiitis obliterans[Buerger] |
| I78.0 | Rendu-Osler-Weber disease |
| I82.0 | Budd-Chiari syndrome |
| J84.0 | Alveolar proteinosis |
| J84.18 | Idiopathic pulmonary fibrosis |
| K50 | Crohn’s disease[regional enteritis] |
| K50.0 | Crohn’s disease of small intestine Crohn’s disease [regional enteritis] of duodenum Crohn’s disease [regional enteritis] of ileum Crohn’s disease [regional enteritis] of jejunum Regional ileitis Terminal ileitis |
| K50.1 | Crohn’s disease of large intestine Granulomatous colitis Regional colitis Crohn’s disease [regional enteritis] of colon Crohn’s disease [regional enteritis] of large bowel Crohn’s disease [regional enteritis] of rectum |
| K50.8 | Other Crohn’s disease Crohn’s disease of both small and large intestine |
| K50.9 | Crohn’s disease, unspecified Crohn’s disease NOS Regional enteritis NOS |
| K51 | Ulcerative colitis |
| K51.0 | Ulcerative (chronic) pancolitis |
| K51.2 | Ulcerative (chronic) proctitis |
| K51.3 | Ulcerative (chronic) rectosigmoiditis |
| K51.4 | Inflammatory polyps |
| K51.5 | Left side colitis |
| K51.8 | Other ulcerative colitis |
| K51.9 | Ulcerative colitis, unspecified |
| K74.3 | Primary biliary cirrhosis Chronic nonsuppurative destructive cholangitis |
| K75.4 | Autoimmune hepatitis |
| L10.0 | Pemphigus vulgaris |
| L10.2 | Pemphigus foliaceus |
| L12.0 | Bullous pemphigoid |
| L12.1 | Cicatricial pemphigoid Benign mucous membrane pemphigoid |
| L12.3 | Acquired epidermolysis bullosa |
| M07.20 | Psoriatic sponlylitis, multiple sites |
| M07.28 | Psoriatic sponlylitis, other(head, neck, ribs, skull, trunk, vertebral column) |
| M08.0 | Juvenile rheumatoid arthritis Juvenile rheumatoid arthritis with or without rheumatoid factor |
| M08.1 | Juvenile ankylosing spondylitis |
| M08.2 | Juvenile arthritis with systemic onset  Still’s disease NOS |
| M08.3 | Juvenile polyarthritis (seronegative) Chronic juvenile polyarthritis |
| M30.0 | Polyarteritis nodosa |
| M30.1 | Polyarteritis with lung involvement [Churg- Strauss] Allergic granulomatous angiitis |
| M30.2 | Juvenile polyarteritis |
| M31.0 | Hypersensitivity angiitis Goodpasture’s syndrome |
| M31.1 | Thrombotic microangiopathy Thrombotic thrombocytopenic purpura |
| M31.2 | Lethal midline granuloma |
| M31.3 | Wegener’s granulomatosis Necrotizing respiratory granulomatosis |
| M31.4 | Aortic arch syndrome [Takayasu] |
| M31.7 | Microscopic polyangiitis |
| M32.1 | Systemic lupus erythematosus with organ or system involvement |
| M32.10 | Systemic lupus erythematosus with endocarfitis Liebman-Sacks disease Systemic lupus erythematosus with pericarditis |
| M32.12 | Systemic lupus erythematosus with lung involvement |
| M32.13 | Systemic lupus erythematosus with tubule-interstitial neophropathy Systemic lupus erythematosus with other organ or system involvement |
| M32.15 | Systemic lupus erythematosus with other organ or system involvement Encephalitis in systemic lupus erythematosus Myopathy in systemic lupus erythematosus Cerebral arteritis in systemic lupus erythematosus |
| M32.19 | Systemic lupus erythematosus with unspecified organ or system |
| M32.8 | Other forms of systemic lupus erythematosus |
| M32.9 | Systemic lupus erythematosus, unspecified |
| M33 | Dermatopolymyositis |
| M33.0 | Juvenile dermatomyositis |
| M33.1 | Other dermatomyositis |
| M33.2 | Polymyositis |
| M33.9 | Dermatopolymyositis, unspecified |
| M34.0 | Progressive systemic sclerosis |
| M34.1 | CR(E)ST syndrome Combination of calcinosis, Raynaud’s phenomenon, (o)esophageal dysfunction, sclerodactyly,  telangiectasia |
| M34.8 | Other forms of systemic sclerosis Systemic sclerosis with lung involvement Systemic sclerosis with myopathy |
| M34.9 | Systemic sclerosis, unspecified |
| M35.0 | Sicca syndrome[Sjögren] Sjogren’s syndrome with keratoconjunctivitiis Sjogren’s syndrome with lung involvement Sjogren’s syndrome with myopathy Sjogren’s syndrome with renal tubulo-interstitial disorder |
| M35.1 | Other overlap syndromes Mixed connective tissue disease |
| M35.2 | Behçet’s disease |
| M35.3 | Polymyalgia rheumatica |
| M35.4 | Diffuse(eosinophilic) fasciitis |
| M35.5 | Multifocal fibrosclerosis |
| M35.6 | Relapsing panniculitis[Weber-Christian] |
| M35.7 | Hypermobility syndrome Familial ligamentous laxity |
| M45 | Ankylosing spondylitis Rheumatoid arthritis of spine |
| M61.1 | Fibrodysplasia ossificans progressiva |
| M88 | Paget’s disease of bone [osteitis deformans] |
| M88.0 | Paget’s disease of skull |
| M88.8 | Paget’s disease of other bones |
| M88.9 | Paget’s disease of bone, unspecified |
| M89.0 | Complex regional pain syndrome type I Algoneurodystrophy Shoulder-hand syndrome Sudeck’s atrophy Sympathetic reflex dystrophy |
| M94.1 | Relapsing polychondritis |
| N25.1 | Nephrogenic diabetes insipidus |
| P22.0 | Respiratory distress syndrome of newborn |
| Q03.1 | Dandy-Walker syndrome |
| Q04.3 | Agyria of brain |
| Q04.6 | Schizencephaly |
| Q05 | Spina bifida |
| Q05.0 | Cervical spina bifida with hydrocephalus |
| Q05.1 | Thoracic spina bifida with hydrocephalus Dorsal spina bifida with hydrocephalus Thoracolumbar spina bifida with hydrocephalus |
| Q05.2 | Lumbar spina bifida with hydrocephalus Lumbosacral spina bifida with hydrocephalus |
| Q05.3 | Sacral spina bifida with hydrocephalus |
| Q05.4 | Unspecified spina bifida with hydrocephalus |
| Q05.5 | Cervical spina bifida without hydrocephalus |
| Q05.6 | Thoracic spina bifida without hydrocephalus Dorsal spina bifida NOS Thoracolumbar spina bifida NOS |
| Q05.7 | Lumbar spina bifida without hydrocephalus Lumbosacral spina bifida NOS |
| Q05.8 | Sacral spina bifida without hydrocephalus |
| Q05.9 | Spina bifida, unspecified |
| Q06.2 | Diastematomyelia |
| Q07.0 | Arnold-Chiari syndrome |
| Q20.0 | Common arterial trunk Persistent truncus arteriosus |
| Q20.1 | Double outlet right ventricle Taussig-Bing syndrome |
| Q20.2 | Double outlet left ventricle |
| Q20.4 | Single ventricle |
| Q21.8 | Eisenmenger’s defect |
| Q22.0 | Pulmonary valve atresia |
| Q22.6 | Hypoplastic right heart syndrome |
| Q23 | Congenital malformations of aortic and mitral valves |
| Q23.0 | Congenital stenosis of aortic valve Congenital aortic atresia Congenital aortic stenosis |
| Q23.1 | Congenital insufficiency of aortic valve Bicuspid aortic valve Congenital aortic insufficiency |
| Q23.2 | Congenital mitral stenosis Congenital mitral atresia |
| Q23.3 | Congenital mitral insufficiency |
| Q23.4 | Hypoplastic left heart syndrome Atresia, or marked hypoplasia of aortic orifice or valve, with hypoplasia of ascending aorta and defective development of left ventricle (with mitral valve stenosis or atresia) |
| Q23.8 | Other congenital malformations of aortic and mitral valves |
| Q23.9 | Congenital malformation of aortic and mitral valves, unspecified |
| Q24.5 | Malformation of coronary vessels Congenital coronary(artery) aneurysm |
| Q25.5 | Atresia of pulmonary artery |
| Q26.0 | Congenital stenosis of vena cava Congenital stenosis of vena cava (inferior)(superior) |
| Q26.1 | Persistent left superior vena cava |
| Q26.2 | Total anomalous pulmonary venous connection |
| Q26.3 | Partial anomalous pulmonary venous connection |
| Q26.4 | Anomalous pulmonary venous connection, unspecified |
| Q26.5 | Anomalous portal venous connection |
| Q26.6 | Portal vein-hepatic artery fistula |
| Q38.3 | Aglossia |
| Q44.2 | Atresia of bile ducts |
| Q64.1 | Exstrophy of urinary bladder Ectopia vesicae Extroversion of bladder |
| Q75.1 | Crouzon’s disease |
| Q75.4 | Mandibulofacial dysostosis Franceschetti syndrome Treacher Collins syndrome |
| Q77 | Osteochondrodysplasia with defects of growth of tubular bones and spine |
| Q77.0 | Achondrogenesis Hypochondrogenesis |
| Q77.2 | Short rib syndrome Asphyxiating thoracic dysplasia[Jeune] |
| Q77.3 | Chondrodysplasia punctata |
| Q77.4 | Achondroplasia Hypochondroplasia Osteosclerosis congenita |
| Q77.5 | Dystrophic dysplasia |
| Q77.6 | Chondroectodermal dyplasia Ellis-van Creveld syndrome |
| Q77.7 | Spondyloepiphyseal dysplasia |
| Q77.8 | Other osteochondrodysplasia with defects of growth of tubular bones and spine |
| Q77.9 | Osteochondrodysplasia with defects of growth of tubular bones and spine, unspecified |
| Q78.0 | Osteogenesis imperfecta Fragilitas ossium Fragilitas ossium |
| Q78.1 | Polyostotic fibrous dysplasia Albright(-McCune)(-Sternberg) syndrome |
| Q78.2 | Osteopetrosis Albers-Schönberg syndrome |
| Q78.4 | Enchondromatosis Maffucci’s syndrome Ollier’s disease |
| Q78.5 | Pyle’s syndrome |
| Q78.6 | Multiple congenital exostoses Diaphyseal aclasis |
| Q79 | Congenital malformations of musculoskeletal system, NEC |
| Q79.0 | Congenital diaphragmatic hernia |
| Q79.1 | Other congenital malformations of diaphragm Absence of diaphragm Congenital malformation of diaphragm NOS Eventration of diaphragm |
| Q79.2 | Exomphalos Omphalocele |
| Q79.3 | Gastroschisis |
| Q79.4 | Prune belly syndrome |
| Q79.5 | Other congenital malformations of abdominal wall |
| Q79.6 | Ehlers-Danlos syndrome |
| Q79.8 | Other congenital malformations of musculoskeletal system Absence of muscle Absence of tendon Accessory muscle Amyotrophia congenita Congenital constricting bands Congenital shortening of tendon Poland’s syndrome |
| Q79.9 | Congenital malformation of musculoskeletal system, unspecified Congenital anomaly of musculoskeletal system NOS Congenital deformity of musculoskeletal system NOS |
| Q81.1 | Epidermolysis bullosa letalis Herlitz’ syndrome |
| Q81.2 | Epidermolysis bullosa dystrophica |
| Q85.0 | Neurofibromatosis (nonmalignant) Von Recklinghausen’s disease |
| Q85.1 | Tuberous sclerosis Bourneville’s disease Epiloia |
| Q85.8 | Sturge-Weber(-Dimitri) syndrome Peutz-Jeghers syndrome Von Hippel-Lindau syndrome |
| Q86.0 | Fetal alcohol syndrome (dysmorphic) |
| Q87.0 | Congenital malformation syndromes predominantly affecting facial appearance Acrocephalopolysyndactyly Acrocephalosyndactyly(Apert) Cryptophthalmos syndrome Cyclopia Goldenhar syndrome Moebius syndrome Oro-facial-digital syndrome Robin syndrome Whistling face |
| Q87.1 | Congenital malformation syndromes predominantly associated with short stature Aarskog syndrome Cockayne syndrome De Lange syndrome Dubowitz syndrome Noonan syndrome Prader-Willi syndrome Robinow-Silverman-Smith syndrome Russel-Silver syndrome Seckel syndrome Smith-Lemli-Opitz syndrome |
| Q87.2 | Rubinstein-Taybi syndrome |
| Q87.3 | Sotos syndrome |
| Q87.4 | Marfan’s syndrome |
| Q90 | Down’s syndrome |
| Q90.0 | Trisomy 21, meiotic nondisjunction |
| Q90.1 | Trisomy 21, mosaicism (mitotic nondisjunction) |
| Q90.2 | Trisomy 21, translocation |
| Q90.9 | Down’s syndrome, unspecified Trisomy 21 NOS |
| Q91 | Edwards’ syndrome and Patau’s syndrome |
| Q91.0 | Trisomy 18, meiotic nondisjunction |
| Q91.1 | Trisomy 18, mosaicism (mitotic nondisjunction) |
| Q91.2 | Trisomy 18, translocation |
| Q91.3 | Edwards’ syndrome, unspecified |
| Q91.4 | Trisomy 13, meiotic nondisjunction |
| Q91.5 | Trisomy 13, mosaicism (mitotic nondisjunction) |
| Q91.7 | Patau’s syndrome, unspecified |
| Q93.4 | Deletion of short arm of chromosome 5  Cri-du-chat syndrome |
| Q93.5 | Angelman syndrome  CATCH22 syndrome |
| Q96 | Turner’s syndrome |
| Q96.0 | Karyotype 45, X |
| Q96.1 | Karyotype 46, X iso(Xq) |
| Q96.2 | Karyotype 46, X with abnormal sex chromosome, except iso(Xq) |
| Q96.3 | Mosaicism, 45, X/46, XX or XY |
| Q96.4 | Mosaicism, 45, X/other cell line(s) with abnormal sex chromosome |
| Q96.8 | Other variants of Turner’s syndrome |
| Q96.9 | Turner’s syndrome, unspecified |
| Q98.0 | Klinefelter’s syndrome karyotype 47, XXY |
| Q98.1 | Klinefelter’s syndrome, male with more than two X chromosomes |
| Q98.2 | Klinefelter’s syndrome, male with 46,XX karyotype |
| Q98.4 | Klinefelter’s syndrome, unspecified |
| Q99.2 | Fragile X chromosome |

Abbreviations: NHIS, National Health Insurance Service; KCD, Korean Standard Classification of Diseases; NOS, Not Otherwise Specified; NEC, Not Elsewhere Classified; ACTH, adrenocorticotropic hormone
