# Supplementary material for: The cumulative incidence and trends of rare diseases in South Korea: a nationwide study of the administrative data from the National Health Insurance Service database from 2011–2015
Source: Orphanet J Rare Dis. 2019 Feb 18;14:49. doi: 10.1186/s13023-019-1032-6 (PMC6379926; doi:10.1186/s13023-019-1032-6)
Supplement: Supplementary file 2 — Table S2. Annual cumulative incidence per 10,000,000 insured population for rare diseases in the register of the co-payment assistance policy according to KCD codes from 2011–2015. Description of data: Additional file 2 includes the annual cumulative incidence per 10,000,000 for each targeted rare disease from 2011–2015. Annual cumulative incidence per 10,000,000 was calculated as the total number of newly enrolled patients with the KCD-7 code in the register of the co-payment assistance policy for rare and incurable diseases during a calendar year, divided by the number of residents with health insurance coverage in each year. (DOCX 80 kb) [file 13023_2019_1032_MOESM2_ESM.docx]

Table S2. Annual cumulative incidence per 10,000,000 insured population for rare diseases in the register of the co-payment assistance policy according to KCD codes from 2011–2015

| KCD | 2011 | 2012 | 2013 | 2014 | 2015 |
| --- | --- | --- | --- | --- | --- |
| A81 | 0.00 | 0.98 | 0.00 | 0.39 | 0.19 |
| A81.0 | 3.73 | 7.04 | 6.80 | 8.11 | 6.73 |
| A81.1 | 0.00 | 0.00 | 0.00 | 0.00 | 0.19 |
| A81.2 | 1.18 | 0.78 | 0.78 | 0.39 | 0.58 |
| A81.8 | 0.00 | 0.20 | 0.00 | 0.00 | 0.19 |
| A81.9 | 0.20 | 0.00 | 0.00 | 0.19 | 0.19 |
| B45 | 0.39 | 0.00 | 0.39 | 0.39 | 0.58 |
| B45.0 | 6.09 | 7.23 | 8.94 | 6.96 | 8.07 |
| B45.1 | 3.34 | 4.69 | 3.11 | 4.25 | 3.07 |
| B45.2 | 0.20 | 0.39 | 0.19 | 0.00 | 0.00 |
| B45.3 | 0.20 | 0.00 | 0.00 | 0.19 | 0.00 |
| B45.7 | 0.00 | 0.00 | 0.19 | 0.39 | 0.19 |
| B45.8 | 0.00 | 0.20 | 0.00 | 0.19 | 0.38 |
| B45.9 | 0.79 | 0.98 | 0.78 | 1.74 | 1.54 |
| D35.2 | 568.67 | 539.39 | 510.22 | 585.43 | 610.37 |
| D55.0 | 1.77 | 0.98 | 0.39 | 0.19 | 0.96 |
| D55.2 | 0.39 | 0.00 | 0.00 | 0.19 | 0.38 |
| D56 | 0.00 | 0.00 | 0.00 | 0.00 | 0.00 |
| D56.0 | 0.00 | 0.00 | 0.19 | 0.19 | 0.00 |
| D56.1 | 1.77 | 1.76 | 1.75 | 1.16 | 2.88 |
| D56.3 | 0.20 | 0.20 | 0.39 | 0.00 | 0.19 |
| D56.4 | 0.00 | 0.00 | 0.19 | 0.19 | 0.00 |
| D56.8 | 0.00 | 0.20 | 0.00 | 0.19 | 0.38 |
| D56.9 | 1.38 | 0.98 | 0.39 | 1.35 | 2.11 |
| D59.5 | 4.32 | 4.89 | 4.47 | 8.31 | 7.11 |
| D60 | 0.39 | 0.20 | 0.00 | 0.39 | 0.77 |
| D60.0 | 0.20 | 0.39 | 0.19 | 0.19 | 0.00 |
| D60.1 | 0.00 | 0.20 | 0.19 | 0.00 | 0.19 |
| D60.8 | 0.39 | 0.39 | 0.39 | 1.74 | 0.19 |
| D60.9 | 4.52 | 4.30 | 7.00 | 8.31 | 5.19 |
| D61.0 | 5.11 | 4.10 | 2.92 | 3.09 | 3.46 |
| D61.2 | 0.20 | 0.39 | 0.97 | 0.97 | 0.38 |
| D61.3 | 14.54 | 16.61 | 21.38 | 19.71 | 20.37 |
| D61.8 | 2.95 | 2.74 | 1.55 | 2.70 | 2.69 |
| D61.9 | 128.07 | 115.69 | 113.32 | 133.12 | 127.80 |
| D64.4 | 0.20 | 0.00 | 0.00 | 0.19 | 0.00 |
| D69.1 | 7.66 | 2.54 | 1.94 | 2.70 | 1.73 |
| D69.30 | 7.66 | 5.67 | 2.72 | 5.02 | 6.92 |
| D69.6 | 125.91 | 101.23 | 91.94 | 94.67 | 85.33 |
| D70 | 44.98 | 48.27 | 43.15 | 39.22 | 33.06 |
| D71 | 2.95 | 2.15 | 0.58 | 2.51 | 0.38 |
| D76.1 | 18.27 | 20.72 | 20.80 | 23.57 | 23.64 |
| D76.3 | 10.02 | 7.04 | 7.19 | 8.11 | 14.61 |
| D80 | 0.39 | 0.00 | 0.39 | 0.00 | 0.00 |
| D80.0 | 0.59 | 0.20 | 0.39 | 3.09 | 0.77 |
| D80.1 | 0.98 | 1.17 | 0.58 | 3.67 | 0.58 |
| D80.2 | 3.14 | 1.56 | 0.78 | 0.97 | 1.73 |
| D80.3 | 3.14 | 3.52 | 4.08 | 5.60 | 9.22 |
| D80.4 | 0.00 | 0.00 | 0.00 | 0.00 | 0.19 |
| D80.5 | 0.00 | 0.00 | 0.00 | 0.58 | 0.00 |
| D80.6 | 0.00 | 0.20 | 0.00 | 0.00 | 0.00 |
| D80.7 | 0.00 | 0.00 | 0.00 | 0.00 | 0.38 |
| D80.8 | 0.20 | 0.00 | 0.00 | 0.00 | 0.19 |
| D80.9 | 0.20 | 0.20 | 0.00 | 0.58 | 0.19 |
| D81 | 0.00 | 0.00 | 0.00 | 0.00 | 0.00 |
| D81.1 | 0.00 | 0.00 | 0.19 | 0.00 | 0.19 |
| D81.2 | 0.00 | 0.00 | 0.00 | 0.19 | 0.00 |
| D81.8 | 0.20 | 0.00 | 0.00 | 0.00 | 0.00 |
| D81.9 | 0.59 | 0.00 | 0.00 | 0.00 | 0.58 |
| D82 | 0.00 | 0.00 | 0.00 | 0.00 | 0.00 |
| D82.0 | 0.00 | 0.39 | 0.58 | 0.39 | 0.19 |
| D82.1 | 1.57 | 0.78 | 1.17 | 3.86 | 3.27 |
| D82.3 | 0.20 | 0.00 | 0.00 | 0.00 | 0.19 |
| D82.4 | 0.59 | 0.39 | 0.00 | 0.39 | 0.19 |
| D82.8 | 0.00 | 0.00 | 0.00 | 0.00 | 0.19 |
| D83 | 0.00 | 0.00 | 0.00 | 0.00 | 0.00 |
| D83.0 | 0.00 | 0.20 | 0.19 | 0.19 | 0.00 |
| D83.9 | 0.00 | 0.59 | 0.19 | 0.39 | 0.38 |
| D84 | 0.00 | 0.00 | 0.00 | 0.19 | 0.00 |
| D84.0 | 0.00 | 0.00 | 0.00 | 0.00 | 0.00 |
| D84.1 | 1.77 | 1.56 | 0.39 | 1.35 | 1.54 |
| D84.8 | 0.20 | 0.00 | 0.00 | 0.00 | 0.19 |
| D84.9 | 0.79 | 0.98 | 0.58 | 0.97 | 0.58 |
| D86 | 4.91 | 4.30 | 3.30 | 2.51 | 4.04 |
| D86.0 | 16.50 | 18.57 | 21.19 | 27.63 | 30.56 |
| D86.1 | 8.84 | 12.31 | 11.27 | 13.33 | 16.53 |
| D86.2 | 6.29 | 7.43 | 8.55 | 11.59 | 10.95 |
| D86.3 | 4.13 | 2.93 | 2.53 | 7.15 | 6.34 |
| D86.8 | 4.71 | 4.10 | 2.33 | 5.22 | 3.84 |
| D86.9 | 25.54 | 23.06 | 24.68 | 30.53 | 29.60 |
| E22.0 | 27.50 | 21.30 | 20.21 | 40.19 | 33.06 |
| E23.0 | 41.64 | 34.00 | 24.49 | 35.94 | 39.97 |
| E24.0 | 3.54 | 2.15 | 2.92 | 7.15 | 3.46 |
| E24.1 | 0.20 | 0.00 | 0.00 | 0.00 | 0.19 |
| E24.3 | 0.00 | 0.59 | 0.00 | 0.19 | 0.00 |
| E25 | 0.59 | 0.59 | 0.97 | 1.35 | 0.58 |
| E25.0 | 9.82 | 10.16 | 7.77 | 23.96 | 12.68 |
| E25.8 | 0.20 | 0.00 | 0.19 | 0.19 | 0.38 |
| E25.9 | 0.79 | 0.00 | 0.78 | 2.70 | 0.77 |
| E27.1 | 7.46 | 4.89 | 4.47 | 10.24 | 6.15 |
| E27.2 | 1.96 | 1.37 | 1.55 | 2.32 | 2.11 |
| E27.4 | 104.89 | 115.30 | 105.74 | 142.20 | 140.68 |
| E34.8 | 1.18 | 1.56 | 0.39 | 1.16 | 1.15 |
| E55.0 | 16.50 | 13.29 | 4.86 | 11.40 | 9.22 |
| E70 | 0.00 | 0.00 | 0.00 | 0.00 | 0.00 |
| E70.0 | 1.96 | 1.37 | 1.94 | 4.25 | 1.35 |
| E70.1 | 2.36 | 2.35 | 1.75 | 2.13 | 2.88 |
| E70.2 | 0.98 | 0.20 | 0.39 | 0.00 | 0.00 |
| E70.3 | 4.91 | 2.35 | 2.53 | 3.67 | 2.31 |
| E70.8 | 0.20 | 0.00 | 0.00 | 0.00 | 0.00 |
| E70.9 | 0.00 | 0.00 | 0.00 | 0.19 | 0.00 |
| E71 | 0.00 | 0.00 | 0.00 | 1.16 | 0.00 |
| E71.0 | 0.00 | 0.59 | 0.58 | 0.19 | 0.19 |
| E71.1 | 1.38 | 1.95 | 2.14 | 2.70 | 1.92 |
| E71.2 | 0.39 | 0.59 | 0.58 | 1.16 | 0.19 |
| E71.3 | 3.73 | 3.71 | 2.53 | 5.60 | 4.42 |
| E72 | 0.20 | 0.39 | 0.00 | 0.00 | 0.58 |
| E72.0 | 2.16 | 1.17 | 1.94 | 1.93 | 1.73 |
| E72.1 | 1.18 | 2.35 | 0.39 | 2.32 | 2.50 |
| E72.2 | 2.16 | 1.56 | 1.94 | 3.67 | 4.42 |
| E72.3 | 0.39 | 0.20 | 0.39 | 0.19 | 0.38 |
| E72.4 | 0.20 | 0.20 | 0.00 | 0.58 | 0.38 |
| E72.5 | 0.98 | 0.59 | 0.19 | 0.39 | 0.38 |
| E72.8 | 0.39 | 0.39 | 0.58 | 0.19 | 0.96 |
| E72.9 | 0.20 | 0.20 | 0.19 | 0.00 | 0.19 |
| E73.0 | 0.00 | 0.00 | 0.00 | 0.00 | 0.00 |
| E73.1 | 0.00 | 0.00 | 0.00 | 0.19 | 0.00 |
| E73.8 | 0.00 | 0.00 | 0.39 | 0.00 | 0.00 |
| E73.9 | 0.20 | 0.39 | 0.39 | 0.00 | 0.19 |
| E74 | 0.00 | 0.00 | 0.00 | 0.39 | 0.19 |
| E74.0 | 3.34 | 2.54 | 3.30 | 6.57 | 5.38 |
| E74.1 | 0.00 | 0.00 | 0.00 | 0.00 | 0.00 |
| E74.2 | 3.73 | 4.10 | 2.92 | 2.70 | 1.92 |
| E74.3 | 0.00 | 0.00 | 0.58 | 0.00 | 0.19 |
| E74.4 | 0.20 | 0.20 | 0.39 | 0.00 | 0.00 |
| E74.8 | 1.96 | 1.17 | 1.55 | 1.16 | 0.77 |
| E74.9 | 0.00 | 0.00 | 0.00 | 0.00 | 0.00 |
| E75.0 | 0.39 | 0.00 | 0.00 | 0.39 | 0.19 |
| E75.1 | 0.00 | 0.00 | 0.00 | 0.00 | 0.38 |
| E75.2 | 2.16 | 6.06 | 2.92 | 7.54 | 7.11 |
| E75.4 | 0.20 | 0.20 | 0.00 | 0.19 | 0.58 |
| E75.5 | 0.98 | 1.95 | 2.14 | 2.70 | 2.88 |
| E76 | 0.00 | 0.00 | 0.00 | 0.00 | 0.00 |
| E76.0 | 0.00 | 0.20 | 0.19 | 0.58 | 0.00 |
| E76.1 | 1.38 | 0.59 | 1.17 | 2.13 | 0.38 |
| E76.2 | 0.79 | 0.59 | 0.39 | 1.16 | 0.19 |
| E76.3 | 0.00 | 0.00 | 0.78 | 0.58 | 0.77 |
| E76.8 | 0.00 | 0.00 | 0.00 | 0.00 | 0.00 |
| E76.9 | 0.00 | 0.00 | 0.00 | 0.00 | 0.00 |
| E77 | 0.00 | 0.00 | 0.19 | 0.00 | 0.00 |
| E77.0 | 0.00 | 0.00 | 0.19 | 1.16 | 0.00 |
| E77.1 | 0.00 | 0.00 | 0.00 | 0.00 | 0.00 |
| E77.9 | 0.00 | 0.00 | 0.00 | 0.00 | 0.00 |
| E79.1 | 0.00 | 0.39 | 0.58 | 0.00 | 0.19 |
| E80.2 | 1.38 | 1.76 | 1.36 | 0.77 | 1.15 |
| E83.0 | 19.05 | 19.35 | 13.99 | 16.23 | 14.41 |
| E83.3 | 4.32 | 4.49 | 5.25 | 9.85 | 5.96 |
| E84 | 0.00 | 0.00 | 0.00 | 0.58 | 0.38 |
| E84.0 | 0.39 | 0.20 | 0.19 | 0.19 | 0.19 |
| E84.1 | 0.20 | 0.20 | 0.00 | 0.00 | 0.00 |
| E84.9 | 0.39 | 0.20 | 0.00 | 0.19 | 0.19 |
| E85 | 1.77 | 2.35 | 0.78 | 2.90 | 2.11 |
| E85.0 | 0.00 | 0.20 | 0.39 | 0.58 | 0.38 |
| E85.2 | 0.00 | 0.00 | 0.19 | 0.00 | 0.77 |
| E85.3 | 0.39 | 0.20 | 0.78 | 0.19 | 0.77 |
| E85.4 | 4.32 | 3.32 | 4.28 | 6.57 | 6.53 |
| E85.8 | 2.36 | 1.95 | 2.14 | 2.32 | 3.07 |
| E85.9 | 11.20 | 16.81 | 15.94 | 19.13 | 17.49 |
| F84.2 | 4.71 | 3.32 | 5.25 | 4.44 | 5.19 |
| G10 | 3.34 | 4.49 | 5.25 | 8.69 | 6.92 |
| G11 | 0.98 | 0.59 | 0.00 | 0.77 | 1.73 |
| G11.0 | 1.57 | 0.39 | 0.39 | 0.58 | 0.38 |
| G11.1 | 9.43 | 8.60 | 5.25 | 13.33 | 14.22 |
| G11.2 | 31.04 | 29.31 | 33.04 | 50.23 | 48.62 |
| G11.3 | 1.18 | 0.98 | 0.19 | 0.58 | 0.38 |
| G11.4 | 11.39 | 8.01 | 7.97 | 12.95 | 11.92 |
| G11.8 | 0.98 | 1.37 | 1.75 | 2.13 | 1.92 |
| G11.9 | 31.82 | 34.00 | 29.35 | 42.51 | 44.20 |
| G12 | 0.79 | 0.78 | 0.78 | 0.77 | 1.73 |
| G12.0 | 0.79 | 0.98 | 0.78 | 0.97 | 0.77 |
| G12.1 | 5.89 | 4.69 | 7.39 | 18.16 | 10.76 |
| G12.2 | 7.86 | 2.54 | 2.14 | 4.44 | 3.07 |
| G12.20 | 1.96 | 1.56 | 1.36 | 2.32 | 2.11 |
| G12.21 | 42.23 | 48.27 | 41.01 | 87.91 | 59.00 |
| G12.22 | 2.36 | 1.76 | 1.17 | 4.44 | 2.69 |
| G12.23 | 5.89 | 4.69 | 5.44 | 8.89 | 4.23 |
| G12.24 | 1.38 | 0.39 | 0.78 | 1.55 | 2.11 |
| G12.8 | 3.73 | 3.52 | 2.53 | 2.51 | 2.88 |
| G12.9 | 5.30 | 4.89 | 4.66 | 10.82 | 4.61 |
| G13 | 0.98 | 0.00 | 0.19 | 0.00 | 0.00 |
| G13.0 | 1.57 | 1.56 | 2.33 | 0.97 | 0.38 |
| G13.1 | 0.59 | 0.39 | 4.08 | 11.59 | 17.68 |
| G13.2 | 0.20 | 0.20 | 0.00 | 0.19 | 0.38 |
| G13.8 | 4.52 | 1.95 | 3.11 | 2.70 | 2.11 |
| G20 | 1884.36 | 1882.58 | 1945.05 | 2164.53 | 2447.80 |
| G23.1 | 38.89 | 17.20 | 17.49 | 37.87 | 24.60 |
| G31.81 | 1.38 | 0.78 | 0.58 | 2.13 | 1.73 |
| G35 | 55.79 | 41.82 | 39.65 | 43.86 | 44.59 |
| G40.4 | . | . | . | 28.02 | 20.18 |
| G40.40 | . | . | . | 70.71 | 34.59 |
| G40.41 | . | . | . | 29.17 | 38.63 |
| G41 | 9.43 | 7.04 | 9.33 | 8.11 | 9.80 |
| G41.0 | 8.25 | 8.79 | 6.61 | 7.15 | 13.64 |
| G41.1 | 0.59 | 0.98 | 0.78 | 2.13 | 2.11 |
| G41.2 | 9.63 | 12.90 | 12.83 | 14.88 | 19.03 |
| G41.8 | 12.38 | 11.73 | 14.77 | 20.29 | 19.22 |
| G41.9 | 89.57 | 110.81 | 125.37 | 142.01 | 152.78 |
| G51.2 | 0.39 | 0.20 | 0.00 | 0.19 | 0.19 |
| G56.4 | 50.09 | 38.70 | 35.18 | 34.58 | 26.33 |
| G60.0 | 38.50 | 30.29 | 27.21 | 26.28 | 29.40 |
| G61 | 2.75 | 3.13 | 1.36 | 1.55 | 2.69 |
| G61.0 | 106.66 | 105.14 | 114.09 | 142.59 | 150.09 |
| G61.1 | 0.20 | 0.00 | 0.00 | 0.19 | 0.00 |
| G61.8 | 15.71 | 13.88 | 15.55 | 17.58 | 15.37 |
| G61.9 | 8.45 | 6.64 | 6.03 | 11.98 | 9.42 |
| G63.0 | 2.36 | 1.56 | 1.75 | 1.55 | 2.50 |
| G70.0 | 121.00 | 115.50 | 112.73 | 180.07 | 160.09 |
| G70.1 | 0.00 | 0.20 | 0.19 | 0.00 | 0.00 |
| G70.2 | 0.00 | 0.20 | 0.00 | 0.19 | 0.00 |
| G71 | 2.95 | 1.17 | 1.94 | 4.83 | 2.69 |
| G71.0 | 38.70 | 34.79 | 27.41 | 59.90 | 39.40 |
| G71.1 | 31.04 | 30.10 | 36.15 | 36.71 | 32.48 |
| G71.2 | 4.32 | 6.25 | 4.47 | 11.21 | 5.96 |
| G71.3 | 12.96 | 16.42 | 12.05 | 10.63 | 9.61 |
| G71.8 | 0.20 | 0.59 | 0.58 | 1.93 | 0.77 |
| G71.9 | 0.98 | 1.37 | 2.33 | 1.74 | 5.19 |
| G90.8 | 1.77 | 3.91 | 2.53 | 2.32 | 2.88 |
| G95.0 | 51.07 | 47.88 | 46.84 | 43.47 | 44.78 |
| H35.31 | 1272.67 | 1207.37 | 1256.01 | 1335.08 | 1726.93 |
| H35.51 | 183.47 | 142.47 | 146.94 | 168.67 | 176.23 |
| H35.58 | 4.91 | 5.86 | 5.64 | 6.96 | 8.26 |
| I27.0 | 49.30 | 44.75 | 32.27 | 43.67 | 34.02 |
| I27.8 | 10.02 | 6.06 | 7.58 | 16.23 | 10.57 |
| I42.0 | 465.34 | 454.57 | 475.23 | 594.70 | 617.09 |
| I42.1 | 36.93 | 36.55 | 34.99 | 54.29 | 48.81 |
| I42.2 | 282.86 | 278.29 | 272.12 | 366.71 | 412.42 |
| I42.3 | 1.18 | 0.39 | 1.17 | 1.55 | 1.15 |
| I42.4 | 3.73 | 1.56 | 2.92 | 3.28 | 4.80 |
| I42.5 | 5.50 | 5.47 | 7.39 | 7.92 | 9.22 |
| I67.5 | 265.77 | 259.14 | 260.07 | 252.91 | 270.78 |
| I73.1 | 71.70 | 54.72 | 44.32 | 47.72 | 45.35 |
| I78.0 | 1.38 | 2.74 | 1.75 | 0.77 | 0.38 |
| I82.0 | 10.41 | 8.21 | 5.64 | 5.80 | 6.53 |
| J84.0 | 7.07 | 6.06 | 2.72 | 4.64 | 3.46 |
| J84.18 | 278.54 | 283.57 | 312.74 | 322.08 | 358.22 |
| K50 | 33.39 | 28.92 | 25.85 | 33.43 | 42.66 |
| K50.0 | 41.84 | 43.58 | 35.96 | 78.44 | 58.04 |
| K50.1 | 41.05 | 42.41 | 39.26 | 85.59 | 60.92 |
| K50.8 | 24.55 | 26.19 | 27.41 | 55.64 | 42.86 |
| K50.9 | 132.39 | 145.01 | 151.02 | 260.83 | 230.62 |
| K51 | 66.39 | 44.95 | 40.82 | 47.72 | 63.42 |
| K51.0 | 63.84 | 46.12 | 47.43 | 92.55 | 85.90 |
| K51.2 | 140.84 | 151.26 | 154.72 | 215.62 | 245.22 |
| K51.3 | 44.79 | 43.78 | 39.65 | 72.26 | 69.57 |
| K51.4 | 0.20 | 0.00 | 0.39 | 0.19 | 0.19 |
| K51.5 | 3.34 | 7.82 | 4.86 | 13.33 | 11.34 |
| K51.8 | 57.75 | 46.32 | 48.79 | 84.63 | 78.99 |
| K51.9 | 267.34 | 254.06 | 263.76 | 576.35 | 505.82 |
| K74.3 | 57.75 | 60.78 | 63.95 | 74.77 | 84.37 |
| K75.4 | 93.11 | 94.98 | 94.66 | 116.89 | 121.65 |
| L10.0 | 14.34 | 10.55 | 12.25 | 14.49 | 18.06 |
| L10.2 | 5.30 | 7.62 | 7.58 | 8.31 | 7.11 |
| L12.0 | 40.27 | 41.04 | 44.12 | 63.18 | 65.53 |
| L12.1 | 0.20 | 0.39 | 0.19 | 0.77 | 0.77 |
| L12.3 | 1.77 | 2.15 | 0.58 | 1.93 | 1.15 |
| M07.20 | 1.18 | 1.95 | 1.94 | 2.32 | 2.88 |
| M07.28 | 0.00 | 0.20 | 0.58 | 0.77 | 0.38 |
| M08.0 | 4.13 | 2.35 | 2.72 | 10.05 | 3.65 |
| M08.1 | 0.39 | 0.20 | 0.39 | 0.58 | 0.77 |
| M08.2 | 0.39 | 0.00 | 0.39 | 0.00 | 0.58 |
| M08.3 | 0.00 | 0.78 | 0.39 | 0.58 | 0.19 |
| M30.0 | 8.64 | 7.62 | 6.03 | 7.92 | 5.19 |
| M30.1 | 9.04 | 8.40 | 8.36 | 12.75 | 10.19 |
| M30.2 | 0.00 | 0.00 | 0.00 | 0.00 | 0.00 |
| M31.0 | 2.36 | 1.56 | 2.72 | 4.06 | 2.88 |
| M31.1 | 7.27 | 5.86 | 7.39 | 8.31 | 7.50 |
| M31.2 | 0.00 | 0.00 | 0.00 | 0.00 | 0.00 |
| M31.3 | 6.68 | 6.84 | 11.08 | 9.27 | 10.57 |
| M31.4 | 26.91 | 20.72 | 23.13 | 31.49 | 28.44 |
| M31.7 | 4.52 | 7.23 | 7.19 | 12.37 | 14.99 |
| M32.1 | 9.43 | 4.10 | 2.33 | 39.03 | 10.76 |
| M32.10 | 0.98 | 1.37 | 1.36 | 14.68 | 5.38 |
| M32.12 | 1.38 | 1.37 | 1.94 | 5.41 | 2.31 |
| M32.13 | 15.13 | 16.42 | 14.97 | 139.30 | 46.32 |
| M32.15 | 12.18 | 11.33 | 11.47 | 44.63 | 21.72 |
| M32.19 | 17.29 | 20.52 | 19.83 | 126.94 | 47.66 |
| M32.8 | 8.45 | 8.21 | 7.39 | 23.96 | 9.42 |
| M32.9 | 158.72 | 162.60 | 158.22 | 658.07 | 275.01 |
| M33 | 0.79 | 0.59 | 0.39 | 1.16 | 0.38 |
| M33.0 | 2.36 | 2.93 | 1.94 | 3.67 | 3.27 |
| M33.1 | 18.86 | 17.78 | 18.66 | 31.69 | 24.79 |
| M33.2 | 16.50 | 15.63 | 19.05 | 28.21 | 19.99 |
| M33.9 | 4.32 | 5.67 | 4.28 | 11.59 | 5.38 |
| M34.0 | 14.54 | 17.00 | 12.05 | 27.24 | 19.22 |
| M34.1 | 4.13 | 2.35 | 2.33 | 4.44 | 4.80 |
| M34.8 | 9.04 | 7.82 | 7.19 | 15.84 | 11.15 |
| M34.9 | 37.91 | 55.89 | 40.82 | 99.31 | 55.92 |
| M35.0 | 217.45 | 227.68 | 220.61 | 264.12 | 279.43 |
| M35.1 | 22.00 | 22.08 | 18.47 | 37.10 | 25.56 |
| M35.2 | 312.91 | 278.49 | 221.97 | 265.86 | 247.72 |
| M35.3 | 43.02 | 42.60 | 60.64 | 67.43 | 67.84 |
| M35.4 | 1.38 | 1.37 | 0.39 | 1.16 | 1.35 |
| M35.5 | 0.00 | 0.20 | 0.00 | 0.00 | 0.19 |
| M35.6 | 0.39 | 0.59 | 0.19 | 0.00 | 0.00 |
| M35.7 | 0.39 | 0.39 | 0.19 | 0.97 | 0.38 |
| M45 | 79.75 | 62.54 | 58.51 | 114.19 | 61.50 |
| M61.1 | 0.79 | 0.78 | 0.19 | 0.77 | 0.00 |
| M88 | 0.39 | 0.00 | 0.00 | 0.58 | 0.19 |
| M88.0 | 0.39 | 0.20 | 0.00 | 0.19 | 0.19 |
| M88.8 | 0.00 | 0.00 | 0.00 | 0.00 | 0.19 |
| M88.9 | 0.20 | 0.00 | 0.19 | 0.39 | 0.00 |
| M89.0 | 26.32 | 11.14 | 10.11 | 10.63 | 9.03 |
| M94.1 | 0.79 | 0.98 | 1.36 | 1.55 | 0.77 |
| N25.1 | 117.07 | 71.72 | 63.17 | 46.18 | 9.99 |
| P22.0 | 335.50 | 459.85 | 520.91 | 580.02 | 700.88 |
| Q03.1 | 5.30 | 3.71 | 5.44 | 5.22 | 4.61 |
| Q04.3 | 2.55 | 1.95 | 3.50 | 2.13 | 2.88 |
| Q04.6 | 8.84 | 7.43 | 8.16 | 9.08 | 7.30 |
| Q05 | 1.96 | 0.78 | 0.39 | 1.55 | 1.92 |
| Q05.0 | 0.39 | 0.20 | 0.00 | 0.00 | 0.00 |
| Q05.1 | 0.00 | 0.00 | 0.19 | 0.00 | 0.00 |
| Q05.2 | 0.20 | 0.59 | 0.58 | 0.58 | 1.35 |
| Q05.3 | 0.39 | 0.59 | 0.00 | 0.00 | 0.19 |
| Q05.4 | 0.00 | 0.59 | 0.39 | 0.97 | 0.00 |
| Q05.5 | 0.20 | 0.00 | 0.19 | 0.00 | 0.19 |
| Q05.6 | 0.20 | 0.39 | 0.00 | 0.19 | 0.00 |
| Q05.7 | 1.57 | 2.15 | 1.94 | 1.93 | 1.15 |
| Q05.8 | 1.18 | 0.98 | 0.39 | 1.35 | 1.15 |
| Q05.9 | 37.91 | 35.37 | 39.65 | 51.97 | 44.78 |
| Q06.2 | 0.79 | 0.20 | 0.78 | 0.58 | 0.19 |
| Q07.0 | 20.63 | 24.23 | 19.44 | 19.71 | 21.52 |
| Q20.0 | 1.38 | 1.76 | 2.92 | 1.55 | 1.15 |
| Q20.1 | 23.18 | 20.52 | 20.60 | 23.57 | 23.45 |
| Q20.2 | 0.00 | 0.98 | 0.39 | 0.97 | 0.77 |
| Q20.4 | 13.16 | 9.58 | 9.72 | 33.62 | 29.02 |
| Q21.8 | 4.13 | 1.95 | 2.14 | 3.86 | 4.04 |
| Q22.0 | 15.32 | 14.85 | 14.19 | 18.55 | 22.68 |
| Q22.6 | 0.79 | 0.78 | 1.36 | 1.74 | 1.92 |
| Q23 | 0.20 | 0.98 | 0.00 | 0.58 | 0.38 |
| Q23.0 | 11.98 | 13.48 | 13.80 | 16.81 | 15.76 |
| Q23.1 | 66.39 | 84.04 | 82.41 | 116.89 | 130.30 |
| Q23.2 | 0.98 | 2.15 | 1.36 | 2.70 | 1.73 |
| Q23.3 | 16.11 | 16.03 | 14.38 | 20.67 | 23.64 |
| Q23.4 | 1.57 | 2.74 | 1.55 | 2.13 | 2.50 |
| Q23.8 | 1.38 | 1.37 | 2.72 | 2.70 | 1.54 |
| Q23.9 | 1.38 | 1.37 | 3.89 | 10.82 | 6.53 |
| Q24.5 | 53.63 | 92.44 | 66.67 | 70.52 | 73.41 |
| Q25.5 | 10.61 | 7.04 | 6.80 | 13.14 | 10.38 |
| Q26.0 | 0.00 | 0.98 | 0.19 | 0.19 | 0.19 |
| Q26.1 | 2.75 | 4.49 | 5.83 | 8.69 | 9.61 |
| Q26.2 | 15.52 | 10.94 | 11.08 | 11.98 | 10.76 |
| Q26.3 | 5.30 | 7.23 | 7.00 | 8.69 | 12.49 |
| Q26.4 | 0.59 | 0.59 | 0.39 | 0.77 | 0.38 |
| Q26.5 | 0.39 | 1.37 | 0.19 | 0.39 | 0.77 |
| Q26.6 | 0.20 | 0.20 | 0.00 | 0.39 | 0.00 |
| Q38.3 | 0.20 | 0.00 | 0.19 | 0.00 | 0.00 |
| Q44.2 | 17.29 | 12.70 | 13.41 | 11.98 | 11.15 |
| Q64.1 | 0.00 | 0.78 | 0.19 | 1.35 | 0.96 |
| Q75.1 | 2.95 | 2.93 | 3.30 | 2.13 | 1.35 |
| Q75.4 | 1.57 | 1.95 | 2.14 | 2.13 | 1.92 |
| Q77 | 0.00 | 0.00 | 0.39 | 0.19 | 0.00 |
| Q77.0 | 0.39 | 0.39 | 0.39 | 0.58 | 0.19 |
| Q77.2 | 0.39 | 0.00 | 0.00 | 0.19 | 0.00 |
| Q77.3 | 1.38 | 1.76 | 1.94 | 1.35 | 1.15 |
| Q77.4 | 10.41 | 9.58 | 8.16 | 8.11 | 8.65 |
| Q77.5 | 0.20 | 0.20 | 0.00 | 0.19 | 0.19 |
| Q77.6 | 0.20 | 0.00 | 0.00 | 0.00 | 0.00 |
| Q77.7 | 2.55 | 2.93 | 2.14 | 3.09 | 1.54 |
| Q77.8 | 4.13 | 3.13 | 2.14 | 1.74 | 4.80 |
| Q77.9 | 0.79 | 0.39 | 0.00 | 0.00 | 1.35 |
| Q78.0 | 10.02 | 6.06 | 6.22 | 6.57 | 8.84 |
| Q78.1 | 5.11 | 4.89 | 4.28 | 4.06 | 6.15 |
| Q78.2 | 1.96 | 2.93 | 1.55 | 1.55 | 0.96 |
| Q78.4 | 6.29 | 5.86 | 5.25 | 5.02 | 4.80 |
| Q78.5 | 0.00 | 0.00 | 0.00 | 0.00 | 0.19 |
| Q78.6 | 5.11 | 2.93 | 7.97 | 6.38 | 2.50 |
| Q79 | 0.39 | 0.20 | 0.39 | 0.00 | 0.00 |
| Q79.0 | 8.45 | 8.40 | 10.88 | 7.73 | 8.65 |
| Q79.1 | 4.13 | 7.23 | 4.47 | 7.15 | 6.73 |
| Q79.2 | 2.55 | 4.30 | 2.53 | 4.44 | 5.00 |
| Q79.3 | 1.57 | 1.17 | 2.14 | 2.90 | 1.35 |
| Q79.4 | 0.00 | 0.20 | 0.58 | 0.19 | 0.19 |
| Q79.5 | 0.20 | 0.20 | 0.78 | 0.39 | 0.58 |
| Q79.6 | 0.59 | 2.35 | 1.17 | 0.97 | 0.58 |
| Q79.8 | 7.07 | 7.23 | 5.44 | 7.92 | 7.69 |
| Q79.9 | 6.29 | 4.89 | 4.86 | 4.44 | 5.38 |
| Q81.1 | 0.20 | 0.00 | 0.00 | 0.00 | 0.00 |
| Q81.2 | 1.96 | 1.17 | 0.19 | 0.77 | 1.15 |
| Q85.0 | 136.32 | 127.23 | 126.73 | 122.11 | 75.53 |
| Q85.1 | 21.61 | 16.81 | 17.49 | 16.81 | 14.03 |
| Q85.8 | 15.52 | 11.92 | 10.30 | 18.35 | 14.80 |
| Q86.0 | 0.00 | 0.00 | 0.00 | 0.00 | 0.19 |
| Q87.0 | 11.00 | 8.79 | 8.36 | 6.57 | 8.65 |
| Q87.1 | 19.05 | 16.81 | 22.35 | 17.78 | 19.03 |
| Q87.2 | 0.98 | 1.17 | 3.11 | 20.48 | 18.45 |
| Q87.3 | 1.77 | 1.95 | 3.30 | 1.74 | 1.54 |
| Q87.4 | 32.21 | 25.99 | 28.38 | 27.82 | 30.75 |
| Q90 | 6.09 | 5.28 | 5.44 | 11.01 | 7.69 |
| Q90.0 | 4.91 | 3.52 | 4.47 | 8.11 | 5.57 |
| Q90.1 | 0.00 | 0.00 | 0.00 | 0.58 | 0.58 |
| Q90.2 | 0.20 | 0.00 | 0.00 | 0.39 | 0.58 |
| Q90.9 | 38.30 | 42.41 | 35.76 | 74.39 | 52.47 |
| Q91 | 0.00 | 0.00 | 0.00 | 0.00 | 0.00 |
| Q91.0 | 0.39 | 0.00 | 0.19 | 0.00 | 0.00 |
| Q91.1 | 0.00 | 0.00 | 0.00 | 0.00 | 0.00 |
| Q91.2 | 0.00 | 0.00 | 0.19 | 0.00 | 0.00 |
| Q91.3 | 0.59 | 0.59 | 0.97 | 0.39 | 0.96 |
| Q91.4 | 0.00 | 0.00 | 0.00 | 0.00 | 0.19 |
| Q91.5 | 0.00 | 0.00 | 0.00 | 0.19 | 0.19 |
| Q91.7 | 0.00 | 0.00 | 0.39 | 0.19 | 0.19 |
| Q93.4 | 1.18 | 2.15 | 4.08 | 1.74 | 1.54 |
| Q93.5 | 10.02 | 7.62 | 8.94 | 15.07 | 11.53 |
| Q96 | 3.54 | 2.35 | 2.33 | 5.02 | 2.50 |
| Q96.0 | 1.96 | 1.76 | 0.58 | 4.06 | 2.31 |
| Q96.1 | 0.20 | 0.20 | 0.00 | 1.16 | 0.58 |
| Q96.2 | 0.00 | 0.00 | 0.00 | 0.19 | 0.00 |
| Q96.3 | 1.18 | 0.98 | 0.39 | 1.74 | 2.11 |
| Q96.4 | 0.39 | 0.20 | 0.19 | 0.77 | 1.15 |
| Q96.8 | 2.95 | 4.10 | 4.86 | 3.86 | 3.84 |
| Q96.9 | 29.07 | 24.62 | 19.44 | 46.76 | 43.05 |
| Q98.0 | 13.95 | 8.99 | 8.75 | 11.79 | 15.76 |
| Q98.1 | 0.39 | 0.59 | 0.58 | 0.77 | 0.77 |
| Q98.2 | 0.00 | 0.59 | 0.19 | 0.39 | 0.58 |
| Q98.4 | 4.32 | 4.49 | 2.72 | 3.48 | 5.96 |
| Q99.2 | 1.18 | 0.20 | 0.78 | 1.16 | 2.88 |

Abbreviations: KCD, Korean Standard Classification of Diseases
